# The revision of baphetids from the Middle Pennsylvanian of the Czech Republic: Morphology, ontogeny, palaeoecology, and the reassessment of the phylogeny of Baphetoidea

**DOI:** 10.1002/ar.70054

**Published:** 2025-09-19

**Authors:** Pavel Barták, Martin Ivanov, Boris Ekrt

**Affiliations:** ^1^ Department of Geological Sciences, Faculty of Science Masaryk University Brno Czech Republic; ^2^ Ostrava Museum Ostrava Czech Republic; ^3^ Department of Palaeontology National Museum Prague Czech Republic

**Keywords:** Carboniferous, development, mandible, Nýřany, skull

## Abstract

The baphetoids represent a clade of the Carboniferous stem‐tetrapods (Middle Mississippian—Middle Pennsylvanian) with a characteristic extension of the orbits into antorbital vacuities, which formed keyhole‐shaped openings on the skull. The more derived baphetids were crocodile‐like piscivores frequently occurring in coal‐bearing lacustrine deposits with abundant fish fauna and known from Central and Western Europe, the United States, and Canada. Several important specimens referred to the group have historically been reported from the late Carboniferous (Moscovian) of the Czech Republic, but the thorough revision and comparison of this material have never been fully undertaken. Here we provide a morphological revision of all available baphetid material from the late Carboniferous of the Czech Republic, including one newly described specimen. The part of the presumably lost type material of *Loxomma bohemicum* was rediscovered and shown here to represent a poorly preserved lower jaw fragment of the temnospondyl *Capetus palustris*, while all remaining material can be referred to *Baphetes orientalis* and provides an important insight into the poorly known baphetid ontogeny. The species can be characterized by the postorbital with a very thin and sharply pointed postfrontal process and a slightly elongate rectangular lateral process of the bone. Other characteristics formerly used to diagnose this species are most likely ontogenetically influenced. The results of the most comprehensive phylogenetic analysis of the Baphetoidea to date indicate that Baphetinae might be polyphyletic, while *“Loxomma” lintonensis* has been recovered outside the clade Loxommatinae and cannot be confidently assigned to the *Loxomma* genus on morphological grounds.

## 1 INTRODUCTION

The baphetoids represent a relatively diverse clade of early stem‐tetrapods with the stratigraphic range extending from the Middle Mississippian (Visean) to the Middle Pennsylvanian (Moscovian), and of which the fossil record is currently limited to a few regions in Central and Western Europe (British Isles, Czech Republic), the United States (Ohio) and Canada (Clack & Milner, 2015). The first baphetoids to be named (Barkas, 1873; Dawson, 1863; Embleton & Atthey, 1874; Hancock & Atthey, 1870; Huxley, 1862; Owen, 1854, 1855) were in fact all members of the less inclusive group possessing large crocodile‐like skulls with the substantial anterior extension of the antorbital vacuities, for which the name Baphetidae Cope, 1875a was first coined, although its priority over the widely used Loxommatidae Lydekker, 1889 was long neglected (Milner & Lindsay, 1998). Thus, three baphetid genera, that is, *Baphetes* Owen, 1854, *Loxomma* Huxley, 1862 and *Megalocephalus* Barkas, 1873, were recognized in the late 19th and early 20th centuries, although the latter genus was not properly distinguished from the second until the seminal work of Watson (1926). The relationship of the baphetids to other basal tetrapods was somewhat unclear around that time, and their members were believed to be related to embolomeres (e.g., Lydekker, 1889; Moodie, 1916; Watson, 1917, 1929). Watson (1929) described a new genus, *Spathicephalus* Watson, 1929, based on three specimens with a distinctive broad and flattened skull, and assigned it to baphetids (=loxommatids), a classification that was followed by other authors (Andrews et al., 1977; Baird, 1962; Romer, 1947). In the same work, he erected a superfamily name Loxommoideae [sic], which had the same content as the family, thus being redundant at the time. In her revision of baphetids, Beaumont (1977) placed *Spathicephalus* on the basis of its distinctive skull morphology in a separate family, Spathicephalidae Beaumont, 1977, thus reinstating the importance of the Loxommatoidea (=Baphetoidea; Milner & Lindsay, 1998) in uniting the two families, with a detailed diagnosis of the group provided later (Beaumont & Smithson, 1998). In this seminal 1977 work, she revised all baphetid species known at that time and described two new taxa, *Loxomma rankini* and *Baphetes lintonensis* (currently ascribed to *“Loxomma” lintonensis*, see Section 3 for explanation of quotation marks). Another possible early baphetoid, *Eucritta melanolimnetes*, has been described by Clack (1998, 2001) and placed at the base of the group (but see Clack et al., 2016, 2019; Ruta et al., 2003 for different hypotheses), showing a mosaic of characteristics shared with the crown tetrapods. In the past two decades, a few other works have concerned themselves with baphetoids to expand the knowledge of the clade (Clack, 2003; Clack & Milner, 2015; Milner et al., 2009; Smithson et al., 2017). Unlike the previous classifications, baphetoids were more recently almost universally considered either to be closely related to temnospondyls (Baird, 1957; Clack, 1998, 2001, 2003; Gauthier et al., 1989; Romer, 1947) or representing a group of stem‐tetrapods (e.g., Clack et al., 2016; Gardiner, 1983; Laurin & Reisz, 1997; Marjanović & Laurin, 2019; Pardo et al., 2017; Ruta et al., 2003), although Panchen (1980) alternatively suggested their position among stem‐amniotes. However, until the publication of Milner et al. (2009), the detailed internal relationships of the group were essentially unknown. In their phylogenetic analysis, *Eucritta* and *Spathicephalus* were recovered as basal‐most baphetoids, and the Baphetidae was divided into two subclades, for which the original names proposed by Lydekker (1889), Baphetinae and Loxommatinae, were reinstated, representing the current systematic concept of the group (Clack & Milner, 2015).

In the late Carboniferous of the Czech Republic, baphetids have so far only been discovered in the coal‐bearing deposits of the Nýřany locality (Middle Pennsylvanian: Moscovian), where their findings are limited to several important specimens. Fritsch (1885) described *Loxomma bohemicum* Fritsch, 1885 based on an isolated fragment of a large dentary with teeth in part and counterpart. This type material was considered indeterminable by Steen (1938), but has not been revised since its original description and was assumed to be lost, representing a *nomen dubium* (Clack & Milner, 2015; Milner et al., 2009). Steen (1938) reported an almost complete articulated skull, the counterpart of which was described by Broili (1908) as *Sclerocephalus credneri*, and referred it to *Loxomma bohemicum*, later transferred to *Baphetes bohemicus* (Fritsch, 1885) by Beaumont (1977). Based on the dubious status of the type specimen (Fritsch, 1885), this dermal skull roof was recently designated as the holotype of a new species, *Baphetes orientalis* Milner et al., 2009, and a well‐preserved juvenile skull with two mandibles and partial pectoral girdle was newly assigned to it (Clack & Milner, 2015; Milner et al., 2009). Here, we provide the comprehensive morphological revision of all baphetid material known from the late Carboniferous of the Czech Republic, including the rediscovered type lower jaw of *Loxomma bohemicum*, as well as one newly described baphetid specimen represented by a well‐preserved isolated dentary with teeth. Consequently, these new morphological data have implications for the taxonomy and ontogeny of the Nýřany baphetids. Furthermore, in order to test a recent hypothesis on the baphetoid interrelationships, we conducted the most comprehensive phylogenetic analysis of the group to date and discuss its implications for the current baphetoid systematics.

## 2 MATERIALS AND METHODS

In this study, all material previously attributed to baphetids from the late Carboniferous of the Czech Republic, as well as one newly assigned specimen, was assembled for the purpose of morphological, systematic, and taxonomic revision. In addition, a mandible referred to the temnospondyl amphibian *Capetus palustris* was used for the anatomical comparison. All samples come from a single lithostratigraphic unit (Main Nýřany Coal Seam, Nýřany Member) of the Kladno Formation in the Pilsen Basin and are preserved on slabs of coal shale either as acid‐etched imprints or light brown colored bone mineralization. A single well‐preserved specimen, ÚGV PAL00220, was subjected to micro‐tomographic imaging using the GE phoenix v|tome|x L240 device at the Central European Institute of Technology in Brno (Czech Republic), with parameters set as follows: the accelerating voltage of 160 kV, the beam current of 250 μA, the voxel resolution of 30 μm and a 0.5 mm Cu filter. The resulting data files were processed with software ITK‐SNAP v.3.8.0 (Yushkevich et al., 2006) to obtain a three‐dimensional surface model.

The phylogenetic analysis was performed in a software PAUP* 4.0a169 (Swofford, 2003), using branch‐and‐bound search with ACCTRAN character optimization. In addition, a bootstrap analysis using branch‐and‐bound search (1000 replicates; retaining groups with frequency > 50%) was conducted to evaluate tree support.

### 2.1 The specimens examined in the present study

NMP M1388 (holotype of *Baphetes orientalis*): acid‐etched imprint of the dorsal skull roof.

NHMW‐Geo‐1898/0010/0042: acid‐etched imprint of the dorsal skull roof, two mandibles, and partial pectoral girdle (examined from the latex peel).

ÚGV PAL00220: isolated right dentary with teeth in lateral view.

NMP M535 (type of *Loxomma bohemicum*): anterior part of the left dentary with teeth in medial view.

NHMW‐Geo‐1898/0010/0051a (referred to *Capetus palustris*): left dentary with teeth in lateral view and anterior portion of a snout.

*Anatomical abbreviations*: a, angular; ang.s.s, angular sutural surface; art.cor, coronoid articulation surface; art.sp, splenial articulation surface; asym, adsymphysial; d, dentary; de.fa, dentary fang; f, frontal; in, internasal; it, intertemporal; j, jugal; l, lacrimal; m, maxilla; Mec.fo, Meckelian fossa; med.sh, medial shelf; mes.psy.for, mesial parasymphysial foramen; n, nasal; nar, naris; nut.for, nutrient foramina; p, parietal; pal, palatine; pf, postfrontal; pmx, premaxilla; po, postorbital; pp, postparietal; prf, prefrontal; psp, postsplenial; pt, pterygoid; q, quadrate; qj, quadratojugal; re.p, replacement pit; res.p, resorption pit; sa.s.s, surangular sutural surface; smx, septomaxilla; sp, splenial; sq, squamosal; sr, surangular; st, supratemporal; t, tabular.

*Institutional abbreviations*: BMNH, Natural History Museum, London, United Kingdom; NHMW, Naturhistorisches Museum Wien, Austria; NMP, National Museum, Prague, Czech Republic; ÚGV PAL, Palaeontological collection of the Department of Geological Sciences, Faculty of Science, Masaryk University, Brno, Czech Republic.

### 2.2 Systematic paleontology

Tetrapoda Goodrich, 1930.

Baphetoidea Cope, 1875a sensu Milner and Lindsay (1998).

Baphetidae Cope, 1875a.

Baphetinae Cope, 1875a sensu Milner et al. (2009).

Genus: *Baphetes* Owen, 1854.

*Type species*: *Baphetes planiceps* Owen, 1854.

*Diagnosis*: Baphetids with skull length up to 320 mm and possessing (1) broad, rounded snout; (2) anteroposteriorly narrow premaxilla bearing 10–11 teeth; (3) large intertemporal (>50% supratemporal length) close to orbital margin; (4) nasal extending anterior to the external naris.

*Baphetes orientalis* Milner et al., 2009.

*Sclerocephalus credneri* Fritsch—Broili, 1908: pl. 1, fig. 2 *non* Fritsch, 1901; Broili, 1908: pl. 1, figs. 1, 3.

*Loxomma bohemicum* Fritsch—Steen, 1938: pp. 237–239, figs. 23, 24; pl. 5, fig. 2 *non* Fritsch, 1885: pl. 58, figs. 3–9.

*Loxomma bohemicus* Fritsch—Romer, 1947: pp. 94–96, fig. 17 *non* Fritsch, 1885.

*Baphetes bohemicus* (Fritsch)—Beaumont, 1977: pp. 89–92, fig. 25.

*Baphetes bohemicus* (Fritsch)—Roček, 1988: p. 516.

*Baphetes bohemicus* (Fritsch)—Štamberg & Zajíc, 2008: p. 167 *non* fig. 248.

*Baphetes orientalis* Milner, Milner and Walsh—Milner et al., 2009: figs. 2–5.

*Baphetes orientalis* Milner, Milner and Walsh—Clack & Milner, 2015: pp. 55–56, fig. 28.

*Holotype*: NMP M1388 (formerly ČGH 3509), the imprint of a nearly complete skull in dorsal view. The counterpart was figured by Broili (1908: pl. 1, fig. 2) and destroyed during World War II (Milner et al., 2009).

*Referred material*: NHMW‐Geo‐1898/0010/0042 (formerly NMW 1898.X.42.), complete skull in dorsal view, two mandibles, partial pectoral girdle and gastralia; ÚGV PAL00220, isolated right dentary with teeth in lateral view.

*Locality and horizon*: Nýřany, 13 km south‐west of Pilsen, West Bohemia, Czech Republic. Main Nýřany Coal Seam, Nýřany Member, Kladno Formation in Pilsen Basin; Middle Pennsylvanian (Moscovian: Asturian).

*Emended diagnosis*: A moderately large baphetid (midline skull length about 180 mm) possessing (1) a very thin, sharply pointed postfrontal process of postorbital inclined at a sharp angle from its antero‐posterior axis (differs from wider, more rounded process with less acute angle in *Baphetes kirkbyi*, *“Loxomma” lintonensis*, *L. acutirhinus* and *L. allmanni*); (2) a slightly elongate, rectangular lateral process of postorbital (shared with *Loxomma allmanni*).

*Remarks*: We agree with the attribution of NHMW‐Geo‐1898/0010/0042 to the juvenile *Baphetes orientalis* (see Milner et al., 2009) based on the two diagnostic characters noted above. The morphological features formerly used by Beaumont (1977) and Milner et al. (2009) to diagnose this species are, however, not shared between adult and juvenile specimens; thus, they are treated here as either ontogenetically variable or dubious.

### 2.3 Description

#### 2.3.1 The holotype

**FIGURE 1:**
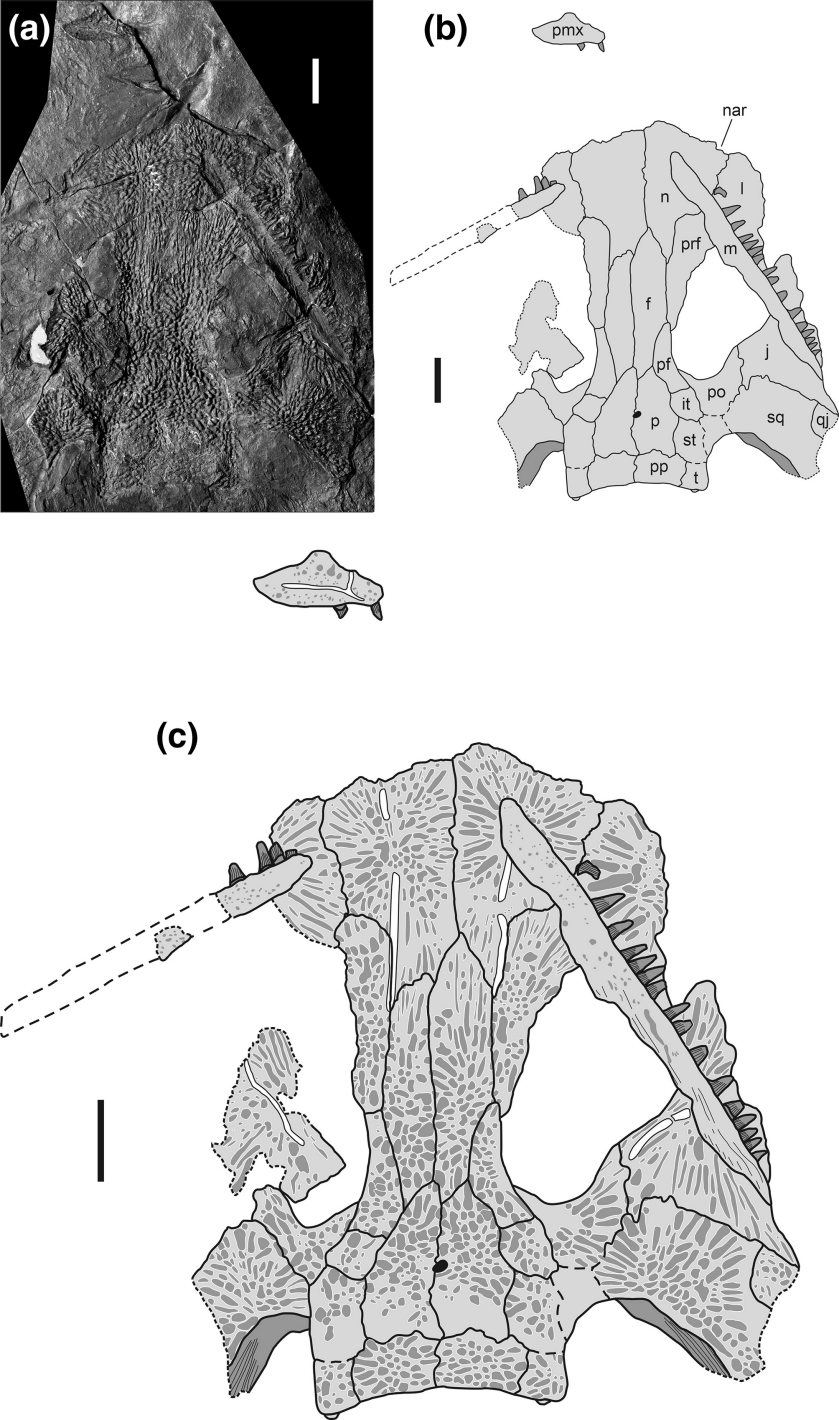
The holotype of *Baphetes orientalis*, NMP M1388, Middle Pennsylvanian, Nýřany, Czech Republic. (a) Photography of the skull roof in dorsal view. (b) Interpretative drawing of the skull with labels of the bones. (c) Interpretative drawing of the skull showing details of the dermal sculpture and lateral line sulci. Scale bars = 20 mm.

NMP M1388 represents the type specimen of *Baphetes orientalis* (Milner et al., 2009) which was previously briefly described by Steen (1938) and Beaumont (1977); the former descriptions of the holotype are expanded and refined in the following section. The specimen is represented by a nearly complete, articulated skull roof impressed on a slab of rock in dorsal view, although the posterolateral cheek region is missing on both sides (Figure 1a–c). Some of the dermal bones have been displaced from their original position (e.g., premaxilla, maxillae), and the left side of the skull appears to be heavily disrupted in the cheek area. The skull measures 180 mm along the midline (including the detached premaxilla), and from its preserved portion, it cannot be discerned whether the cheek region significantly extended posterior to the skull table. The dermal skull roof bears coarse ornamentation consisting of deep pits and elongated ridges and grooves on most of the bones, indicating a possible adult ontogenetic stage of the individual (Figure 1a,c). The posterior border of the small external naris can be seen at least on the right side of the skull and is formed by the nasal and lacrimal. The orbit is rounded and placed behind the mid‐length of the skull. The antorbital fenestra is exposed on the right side only; it is extensive, subrectangular, and longer than the orbit, although its anterior margin is obscured by the displaced right maxilla. The temporal notch is preserved on both sides of the skull, but the width of the right one is probably exaggerated due to deformation of the cheek region caused by the dorsoventral compression. The lateral line system is formed by elongate sulci and present on the premaxilla, nasal, and jugal (Figure 1c). We cannot confidently identify the presence of the lateral line grooves either on the frontal, postfrontal, and postorbital depicted by Beaumont (1977) or on the maxilla and prefrontal shown by Steen (1938).

The left premaxilla is separated from the skull roof and rotated 180°. It has a subtriangular shape but is probably incomplete to some degree. The external surface bears a Y‐shaped lateral line sulcus and numerous small foramina (Figure 1a,c). The two incomplete teeth are preserved, but the total premaxillary tooth number cannot be determined. The maxilla is an elongate tooth‐bearing element that is completely preserved on both sides of the skull (the extent of the left maxilla is not fully shown in Figure 1, but see Beaumont, 1977 and Steen, 1938). It is rounded anteriorly and gradually tapers in the posterior direction to a short, pointed termination, which bears striated horizontal lamina to receive the jugal (Figures 1a–c and 3a,b). Although the right maxilla is slightly medially displaced, it can be recognized from its posterior extent it did not contact the quadratojugal, and it was excluded from the ventral margin of the antorbital fenestra by the lacrimal‐jugal contact. The right maxilla has 16 teeth preserved, and there would be a space for about 30 tooth positions in a complete dentition. The teeth are conical and have fine longitudinal grooves at the base (Figure 3a,b). In the left maxilla, the anterior‐most part bearing about four incomplete teeth is preserved, while the remaining portion of the bone is present only in the form of an outline without details of the superficial structure. The nasals are large, plate‐like bones forming most of the snout region. The anterior margin of the nasal is beveled at the contact with the premaxilla, while laterally the bone contributes to the medial margin of the external naris and sutures with the lacrimal posteriorly to exclude the maxilla from its posterior border. The posterolateral portion of the nasal is confined by the prefrontal and forms a pointed projection wedged between the prefrontal and the anterolateral border of the frontal. The supraorbital sulci of the lateral line system pass through the central part of the nasals and posteriorly along the nasal‐prefrontal suture, but do not extend on the frontals (Figure 1c). Both lacrimals are partially covered by the maxilla, and the left one appears to be incomplete posteriorly, making it difficult to infer the original shape of the bone. It seems to be rounded and wide anteriorly, contributing to a very broad snout area. The posteromedial suture with the prefrontal appears to be short on the left side, while it is hidden by the overlying maxilla on the right. In the better preserved right lacrimal, the posterior projection of the bone can be seen to contact the anterior process of the jugal. The prefrontal is an elongated element contributing to the medial border of the antorbital fenestra. In contrast to Beaumont (1977) and more similar to the interpretation of Steen (1938), the anterior part of the bone appears to be fairly extensive and invades the posterolateral portion of the nasal. The left prefrontal is posterolaterally, close to the point‐like suture with the postfrontal, slightly bulged to delimit the anteromedial edge of the orbit, while the right prefrontal appears to be nearly straight in that region. The paired frontal is a narrow and elongate bone forming the main component of the interorbital region. According to our interpretation, the anterior part of the frontal is not uniformly developed on both sides, but the right element is longer and wedges between the nasals, whereas the left one contacts the posterior part of the corresponding nasal in a short oblique suture; this is consistent with the reconstruction of Steen (1938) and similar to the condition seen in *Baphetes planiceps* and *B. kirkbyi* (Beaumont, 1977). The frontal is laterally excluded from the orbital margin by the prefrontal‐postfrontal contact, and its posterior part is laterally slightly constricted by the postfrontal and wedges between it and the parietal. The postfrontal is a small crescent‐like bone forming most of the medial border of the orbit and reaches slightly more than half the length of the prefrontal (Figure 1a–c). It has a convex suture with the frontal and parietal medially, and its slightly expanded posterior part contacts the intertemporal posteriorly and the anteromedial process of the postorbital posterolaterally.

The jugal is a large bone of the cheek region which contributes to the posterolateral margin of the antorbital fenestra. It is well‐preserved on the right side, whereas a small portion of the left jugal has been displaced into the orbit and is heavily fractured (Figure 1a–c). The anterior process of the right jugal is partly obscured by the displaced maxilla, but it can still be seen it is stout and contacts the posterior process of the lacrimal. The posterior part of the jugal is extensive and subtriangular in shape. It has a subquadrangular medial projection contacting the lateral process of the postorbital and a bulged anterior margin that defines the boundary between the orbit and the confluent antorbital fenestra. However, the bulging area is not formed solely by the jugal, as suggested by Beaumont (1977) and formerly used as a diagnostic character of the species (Milner et al., 2009), but the postorbital appears to be also involved in its construction, thus being more consistent with the original interpretation of Steen (1938). Posteromedially, the jugal contacts the squamosal along an oblique suture and joins the quadratojugal in a pointed termination. An elongate sulcus of the lateral line canal runs along the anterior margin of the bone on both sides of the skull (Figure 1c). The postorbital is a crescent‐like bone well‐preserved on each side of the skull. It connects the jugal laterally and participates in the formation of a bulging area constricting the lateral border of the orbit to create a characteristic “key‐hole” shaped opening. Posteriorly, the bone wedges between the squamosal laterally and the supratemporal medially, whereas the medial border is almost straight and sutures with the intertemporal. The postorbital forms a very slender, pointed anteromedial process to contact the postfrontal at an acute angle from its antero‐posterior axis. The intertemporal is a relatively small quadrangular bone of the skull table which is surrounded by the postfrontal anteriorly, the parietal medially, the supratemporal posteriorly, and the postorbital laterally. In contrast to *Loxomma*, the intertemporal reaches more than 50% of the supratemporal length and is placed relatively close to the orbit (Figure 1a–c). The supratemporal is present on both sides of the skull table, although neither seems to be completely preserved. The right supratemporal lacks the lateral portion of the bone, while the left one shows a rectangular shape that might result from the squashed cheek area under the skull table. If completely preserved, the supratemporal would likely have a lateral projection contacting the squamosal and contributing to the anterior margin of the temporal embayment present in most other baphetids. The parietal is a roughly subrectangular bone that occupies much of the skull table dermal surface and reaches a little more than half the length of the frontal bone. The anterior part of the element is narrowly pointed and wedges between the frontals on the left and the frontal and the postfrontal on the right. Laterally, the parietal contacts the postfrontal, intertemporal, and supratemporal, and posteriorly it sutures with the postparietal. The small parietal foramen is placed at the midpoint of the interparietal suture and is flush with the skull table (Figure 1a,c). The paired postparietal forms the posterior border of the skull table. It is wider than long and its width exceeds the posterior border of the parietal, resulting in the anterolateral edge joining with the supratemporal that prevents the parietal‐tabular suturing. The tabular represents a small rectangular bone of the posterolateral part of the skull table, which contacts the supratemporal anteriorly and the postparietal medially. A small unornamented knob‐like horn projects from the posterolateral subdermal part of the element (Figure 1b,c). The squamosal is a large crescentic bone forming much of the cheek region and contributing significantly to the anterior and lateral border of the temporal embayment. The posterolateral part of the bone is broken off on both sides of the skull, thus its original extent behind the skull table cannot be determined. Moreover, the left squamosal is probably slightly compressed underneath the lateral skull table, resulting in mediolateral compaction of the temporal embayment. The squamosal flange is well‐developed on both sides and bears longitudinal striation on its surface (Figure 1a,c). On the right side, the suture with the postorbital and partly the jugal is strongly interdigitated. Only a small anterior‐most portion of the right quadratojugal is preserved. It contacts the posterior process of the jugal anteriorly and the squamosal medially. The presence of a partial pterygoid bone on the left side of the skull, documented by Beaumont (1977), can be confirmed here (not figured). It is exposed in ventral view and shows denticle shagreen.

#### 2.3.2 NHMW‐Geo‐1898/0010/0042

**FIGURE 2:**
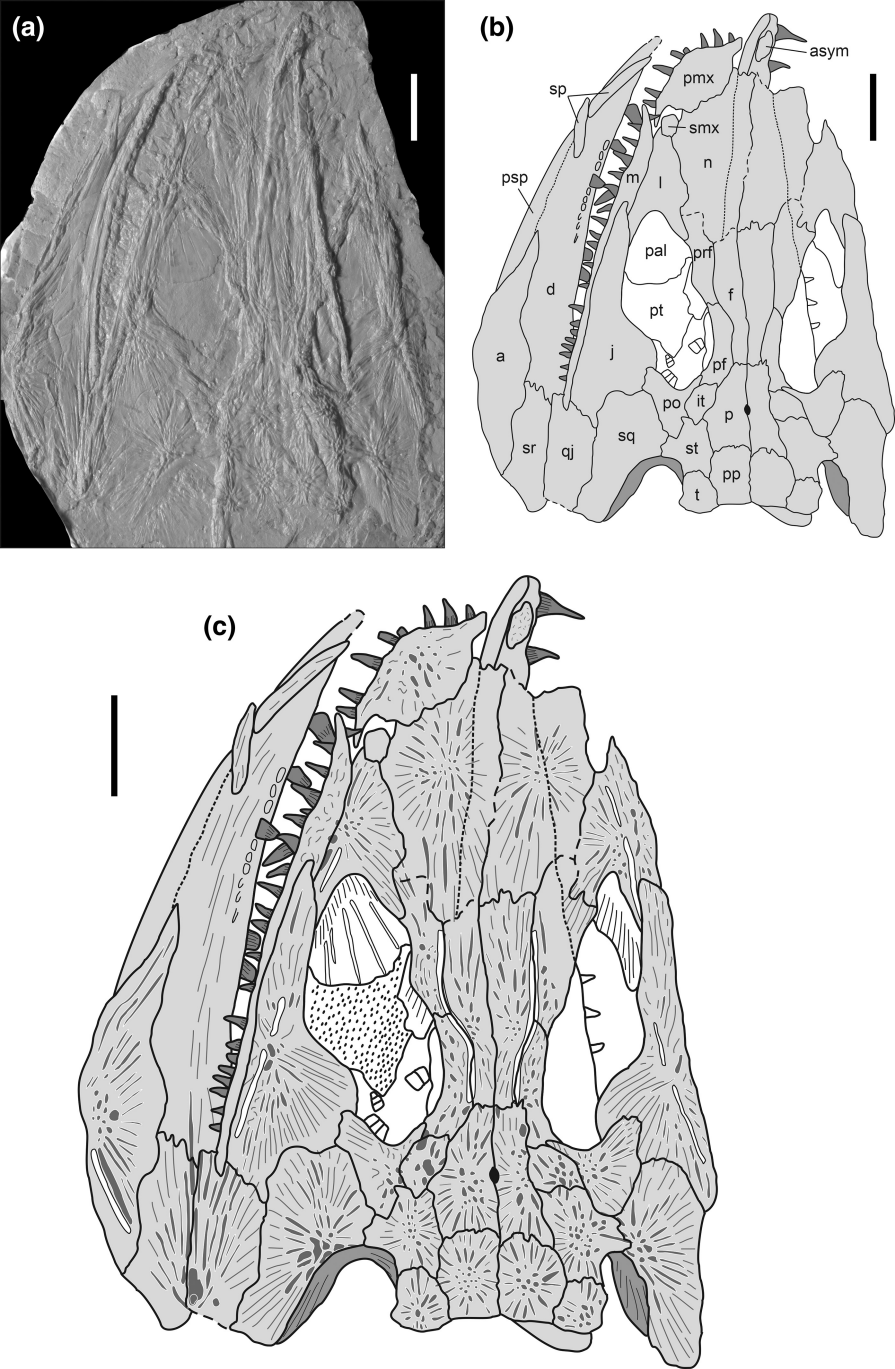
The specimen referred to *Baphetes orientalis*, NHMW‐Geo‐1898/0010/0042, Middle Pennsylvanian, Nýřany, Czech Republic. (a) Photography of the skull in dorsal view with associated mandibles. (b) Interpretative drawing of the skull and mandibles with labels of the bones. (c) Interpretative drawing of the skull and mandibles showing details of the dermal sculpture and lateral line sulci. The associated bones of the pectoral girdle were omitted in the specimen. Scale bars = 10 mm.

The specimen NHMW‐Geo‐1898/0010/0042 has been thoroughly described by Milner et al. (2009) and the morphological information contained in that work will not be repeated here. We focus our description on the differences in anatomical interpretations and details not covered by the previous authors. The dermal sculpture present on the skull roof consists of the central pits and the radiating ridges and grooves on most of the bones, indicating a juvenile stage of the specimen (Figure 2a–c). The ossification center is located at the bone mid‐length in the nasal, frontal, parietal, postparietal, tabular, lacrimal, jugal, postorbital, and supratemporal, while the intertemporal shows the densely arranged pits on most of its surface. In the squamosal, the pits are concentrated close to the anterior margin of the temporal embayment, whereas the quadratojugal has a center of ossification located near its posterior border. The prefrontal extends anterior to the frontal margin and invades the posterolateral border of the nasal to contact it and the lacrimal in a rounded suture. We cannot confirm the presence of a slender and elongate lacrimal process of the prefrontal depicted by Milner et al. (2009), although a short anterolateral projection can be seen on the right side. The lacrimal process of the right jugal does not project far forward of the anterior margin of the antorbital fenestra but terminates on the same level with it, as can be seen on the left side of the skull (Figure 2a–c). The lateral process of the left postorbital does not extend into the jugal in an elongate triangular projection but rather has the form of a short rectangle, as present in the holotype of *B. orientalis* and *Loxomma allmanni* (Beaumont, 1977). In addition, the postfrontal process of the left postorbital appears to be more slender and pointed, similarly to the right postorbital. We found no clear evidence on the presence of a lateral line sulcus on this bone (Figure 2c).

**FIGURE 3:**
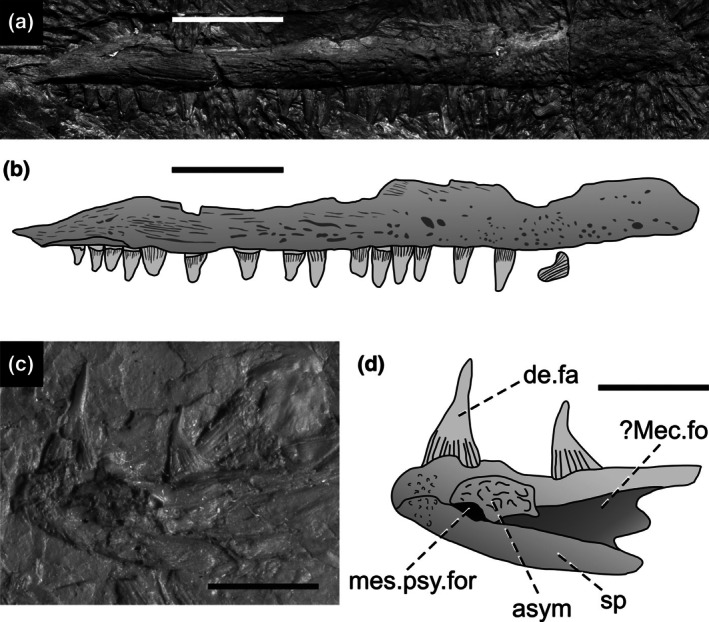
Details of the jaw elements of *Baphetes orientalis*, Middle Pennsylvanian, Nýřany, Czech Republic. (a, b) NMP M1388 (holotype), right maxilla from the lateral view depicted as photography (a) and the interpretative drawing (b). (c, d) NHMW‐Geo‐1898/0010/0042, anterior portion of the right mandible in medial view depicted as photography (c) and the interpretative drawing (d). Scale bars = 20 mm (a, b), 5 mm (c, d).

The lateral surface of the left angular is well exposed in the posteroventral part of the mandible (Figure 2a–c). Unlike most other baphetids (Beaumont, 1977), it possesses an elongate anterior process which wedges between the postsplenial ventrally and the dentary dorsally. The dermal ornamentation consists of elongate ridges and grooves radiating from the ossification center located near the ventral margin at mid‐length of the bone. An elongate lateral line sulcus runs parallel to the dermal ornamentation in the bone's posterior part (Figure 2c). The symphysial region of the right mandible is well exposed in medial view in NHMW‐Geo‐1898/0010/0042, providing some details on its morphology (Figure 3c,d). The mandible has a rounded and relatively shallow anterior portion that bears a roughened surface left by a ligament impression, resembling the condition seen in *Megalocephalus pachycephalus* (Ahlberg & Clack, 1998; Beaumont, 1977). An elongate mesial lamina of the splenial is exposed on the anteroventral margin of the jaw and extends slightly posteriorly to the anterior termination of the mandible. We interpret the roughened plate‐like structure in the symphysial region as the actual adsymphysial plate, since it has similar dimensions and location to that described by Ahlberg and Clack (1998) in *Megalocephalus pachycephalus*. In contrast to *Baphetes kirkbyi* (Milner & Lindsay, 1998), *Spathicephalus mirus* (Beaumont & Smithson, 1998) and the “Parrsboro jaw” (Godfrey & Holmes, 1989; Sookias et al., 2014), the adsymphysial plate of NHMW‐Geo‐1898/0010/0042 lacks any fangs or dentition row (shared with *Kyrinion martilli*; Clack, 2003). A small foramen is identified at the border between the splenial and adsymphysial plate at the posterior level of the enlarged anterior dentary tooth (Figure 3c,d). It could correspond to the foramen figured by Ahlberg & Clack, 1998, fig. 18C in *Megalocephalus pachycephalus*, but unlike these authors, we tentatively consider this opening to be homologous with the mesial rather than lateral parasymphysial foramen of some other stem‐tetrapods and tetrapodomorphs, given its position ventromedial to the adsymphysial bone. The triangular anterior portion of the Meckelian fossa might be exposed posterior to the adsymphysial plate, although this area is poorly preserved in NHMW‐Geo‐1898/0010/0042. Milner et al. (2009) noted the absence of “pseudocanine peaking” along the maxilla and dentary. Although enlarged marginal teeth distributed along the dentary length, seen in *Crassigyrinus scoticus* and *Megalocephalus pachycephalus* (Beaumont, 1977; Panchen, 1985; Porro et al., 2023), are indeed absent in NHMW‐Geo‐1898/0010/0042, the marginal dentition of this specimen exhibits the usual baphetid condition with the dentary fangs present at the symphysis (Figures 2 and 3c,d). The base of the dentary fang has approximately the same width as that of the following tooth (2.5 mm), but the former is about 30% longer than the latter. The upright apical portion of the dentary fang is elongate, slender, and laterally compressed, forming mesial and distal carinae, thus conforming to the “lanceolate” tooth pattern characteristic of other baphetids (Clack & Milner, 2015). It is not possible to discern from the state of preservation whether the dentary fang is placed mesial to the other marginal teeth as in *Baphetes kirkbyi* and *Megalocephalus pachycephalus* (Beaumont 1977; Milner & Lindsay, 1998), or is aligned with the tooth row. All teeth in the upper and lower jaws bear longitudinal grooves on the base. The pectoral girdle elements have been accurately described by Milner et al. (2009).

#### 2.3.3 ÚGV PAL00220

**FIGURE 4:**
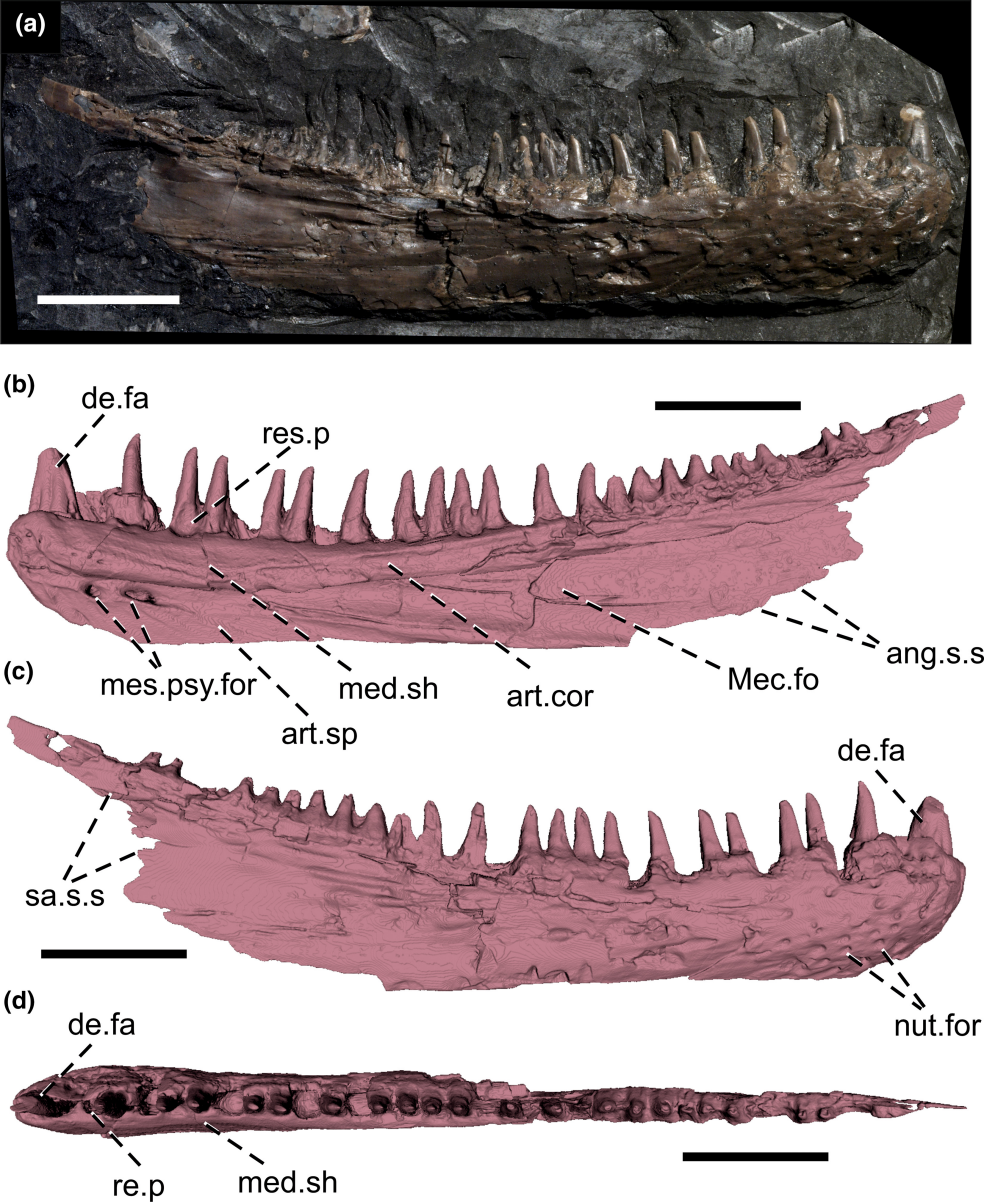
The isolated right dentary referred to *Baphetes orientalis*, ÚGV PAL00220, Middle Pennsylvanian, Nýřany, Czech Republic. (a) Photography of the specimen in lateral view. (b–d) Micro‐computed tomography of the specimen in medial (b), lateral (c) and dorsal (d) views. Scale bars = 10 mm.

**FIGURE 5:**
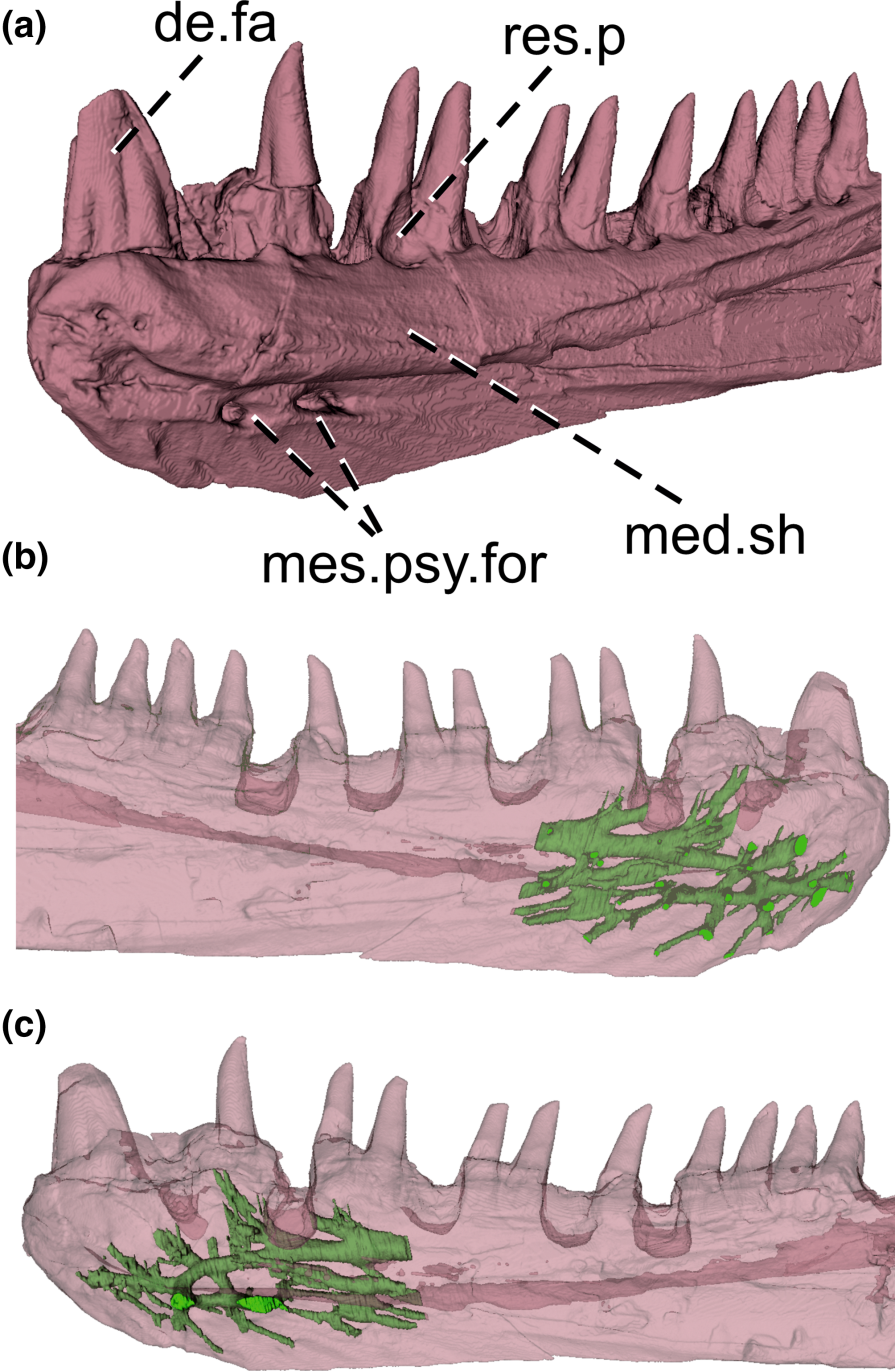
Details of the symphysial region and neurovascular system in the specimen referred to *Baphetes orientalis*, ÚGV PAL00220, Middle Pennsylvanian, Nýřany, Czech Republic. (a) dentary from the anteromedial view. (b,c) Details of the neurovascular system from lateral (b) and dorsomedial (c) views.

The specimen ÚGV PAL00220 represents a complete, isolated right dentary embedded in the rock matrix in lateral view and reaching 65 mm in length (Figures 4a–d and 5a–c). The micro‐CT processing enabled the exposure of the medial surface in the jaw fragment, which would otherwise remain hidden in the surrounding matrix. The symphysial region of the dentary is broadly rounded and bears a thin sheet of bone covering the tooth bases in the lateral view and continuing posteriorly along the tooth row, although being disrupted in several places. The dentary is slightly constricted behind the symphysis, having straight, subparallel dorsal and ventral borders. The posterior portion of the bone is dorsoventrally expanded and forms an oblique ventral margin for connection with the anterior process of the angular, as well as a slender, elongate dorsal process elevated above the tooth row. The latter forms a step‐like margin to connect the anterior part of the surangular in an L‐shaped suture. The lateral surface of the symphysial region bears numerous nutrient foramina for nerves and blood vessels (Figures 4a,c and 5b), while most of the dentary surface is smooth with only a few fine ridges present in the posterior part of the bone. In medial view, a prominent, rounded medial shelf extends at an oblique angle ventrally from the dorsal border of the dentary to approximately half the height of the bone in its anterior part (Figures 4b,d and 5a). More posteriorly, the shelf is flattened and bears a concave medial surface for tongue‐and‐groove articulation with the coronoid bones. The anteroventral part of the dentary has an elongate and narrow articulating surface for the mesial exposure of the splenial. In the anteromedial part, at the sutural border between the dentary and the splenial, two foramina are situated (Figures 4b and 5a). Ahlberg and Clack (1998) reported an opening in a similar position in *Megalocephalus* and hypothesized it might be homologous to the lateral parasymphysial foramen of some other early tetrapods. However, ÚGV PAL00220 exhibits a peculiar condition in which two foramina are present instead of the usual one. The micro‐CT slices demonstrate that these foramina are connected to the mandibular canal (Figure 5c), which likely transmitted the mandibular branch of the trigeminal nerve and artery, similarly to the openings present in some tetrapodomorphs (Clément & Lebedev, 2014; Lebedev & Clément, 2019). Thus, we tentatively consider the foramina present in ÚGV PAL00220 to be structurally homologous to the mesial parasymphysial foramen (sensu Ahlberg, 1995) of some basal tetrapods and tetrapodomorphs. Posterior to these foramina, a large triangular Meckelian fossa occupies most of the medial surface of the dentary and is delimited by the medial shelf dorsally and a subtle ridge running parallel to the sutural surface of the splenial ventrally.

The marginal dentition is well‐preserved throughout the jaw fragment and consists of 22 implanted teeth with space for a total of about 32 teeth. A large dentary fang aligned with the tooth row is present in the anterior‐most part of the jaw fragment, but about its apical third is broken off. The base of the fang is one‐third wider compared to the regular width of other teeth, although the breadth of the subsequent tooth has similar proportions (Figure 5a). The dentary fang, when completely preserved, would probably have slightly exceeded the length of the anterior teeth. A poorly preserved replacement pit is present behind the dentary fang (Figure 4d). The marginal teeth are distinctly long and slender relative to the jaw ramus, but their length rapidly decreases behind the dentary mid‐length. The teeth are upright and have rounded bases with longitudinal grooves almost entirely hidden by a bony sheet in lateral view, whereas their apical half is smooth and flattened to form gently developed carinae. Some teeth (e.g., third, eighth, and tenth) show suboval resorption pits at the lingual base (Figures 4b and 5a), indicating an active tooth replacement.

*Remarks*: ÚGV PAL00220 displays primitive characteristics shared with stem‐tetrapods, such as anterior dentary fangs and possible mesial parasymphysial foramina (Ahlberg & Clack, 1998). Among them, the greatest resemblance is recorded with *Crassigyrinus scoticus* (Panchen, 1985; Porro et al., 2023) and those baphetids for which reasonable dentary material is known, including *Megalocephalus pachycephalus*, *“Loxomma” lintonensis*, *Baphetes orientalis*, *Baphetes kirkbyi*, and *Kyrinion martilli* (Ahlberg & Clack, 1998; Beaumont, 1977; Clack, 2003; Milner et al., 2009; Milner & Lindsay, 1998; Romer, 1930). The general resemblance of the lower jaw morphology between *Crassigyrinus* and baphetids was already noticed by Lydekker (1890) and Panchen (1973), and it led both authors to the conclusion that one of the best preserved mandibles of the former, originally described as *Macromerium scoticum* (Panchen, 1985), belonged to this group. Interestingly, *Crassigyrinus* has been recently recovered in close relationships with baphetoids (Smithson et al., 2024). ÚGV PAL00220 shares with *C. scoticus*, *M. pachycephalus*, and *“L.” lintonensis* a broadly rounded symphysial region, relatively narrow central part of dentary with nearly horizontal ventral margin, and an elongate and slender posterodorsal process for articulation with the surangular (Beaumont, 1977; Panchen, 1985; Porro et al., 2023). Additional shared characteristics between this specimen and *C. scoticus* and *“L.” lintonensis* include deep nutrient foramina in the symphysial region and faint lateral dermal sculpture consisting of only a few subparallel ridges. The morphology and arrangement of the dentition in ÚGV PAL00220 correspond to other baphetids; the teeth are covered at the base by a thin sheet of bone in lateral view, as described in *Megalocephalus pachycephalus* (Beaumont, 1977; Embleton & Atthey, 1874), and the estimated total number of 32 teeth in the lower jaw of the specimen is most similar to *“Loxomma” lintonensis* (35) and *Megalocephalus pachycephalus* (34), but differs from at least 38 tooth positions present in *Crassigyrinus scoticus* (Panchen, 1985; Porro et al., 2023). The dentary tooth crowns show the distinctive lanceolate pattern characterizing baphetids, having a suboval base and laterally compressed apex to form prominent carinae (Clack & Milner, 2015), the condition documented in *Megalocephalus* (Embleton & Atthey, 1874), *Kyrinion* (Clack, 2003), *“Loxomma” lintonensis* (Romer, 1930), *Baphetes kirkbyi* (Milner & Lindsay, 1998) and *Baphetes orientalis*. These comparisons therefore indicate that ÚGV PAL00220 can be attributed to Baphetidae.

Among baphetids, only limited comparison is possible due to the lack of well‐preserved mandibles in most of the species. ÚGV PAL00220 can be clearly distinguished from *Megalocephalus pachycephalus* by the absence of enlarged teeth distributed along the dentary length and by the slightly lower tooth number (Ahlberg & Clack, 1998; Beaumont, 1977). In contrast to *Megalocephalus pachycephalus* and *Baphetes kirkbyi* (Beaumont, 1977; Milner & Lindsay, 1998), the anterior dentary fangs of ÚGV PAL00220 are aligned with the tooth row and are not placed on the distinct horizontal shelf mesial to it. *“Loxomma” lintonensis* differs from the latter in having two enlarged anterior dentary teeth at the same time (Beaumont, 1977; Romer, 1930), while a possible replacement pit is present posterior to the dentary fang in ÚGV PAL00220. We found almost no distinguishing features between ÚGV PAL00220 and NHMW‐Geo‐1898/0010/0042 (referred herein to the juvenile *Baphetes orientalis*, see also Milner et al., 2009), although the comparisons are limited. In both specimens, the symphysial region is relatively shallow and rounded in medial view, and the mesial lamina of the splenial is well exposed anteroventrally. A single parasymphysial foramen is recognized in NHMW‐Geo‐1898/0010/0042, contrary to ÚGV PAL00220, in which two foramina are present, although the former has an articulated adsymphysial plate obscuring details of the region. The more anterior parasymphysial foramen is, however, placed at the posterior level of the dentary fang in both specimens. The dentary fang has the same width at the base as the following tooth in NHMW‐Geo‐1898/0010/0042, but is only about a third longer relative to it. In ÚGV PAL00220, the same width of these two teeth can also be observed, while the apico‐basal length of the dentary fang is unclear. However, its preserved portion indicates that about one‐third of its apical part might be missing, suggesting similar proportions to those in NHMW‐Geo‐1898/0010/0042. Considering the fact that a single baphetid species is currently known from the Middle Pennsylvanian of the Nýřany locality, and based on the shared characteristics with NHMW‐Geo‐1898/0010/0042 discussed above, we tentatively refer the isolated dentary of ÚGV PAL00220 to *Baphetes orientalis*.

Temnospondyli Zittel, 1888.

Genus: *Capetus* Steen, 1938.

*Type species*: *Capetus palustris* Steen, 1938.

*Diagnosis*: As for the type and only species.

*Capetus palustris* Steen, 1938.

*Loxomma bohemicum* Fritsch—Fritsch, 1885: p. 16, pl. 58, figs. 3–9 *non* Steen, 1938: figs. 23, 24; pl. 5, fig. 2.

*Sclerocephalus credneri* Fritsch—Broili, 1908: pl. 1, figs. 1, 3 *non* fig. 2.

*Chelydosaurus vranii* Fritsch—Jaekel, 1911: fig. 124 *non* Fritsch, 1885.

*Capetus palustris* Steen—Steen, 1938: pp. 241–242, fig. 27.

*Gaudrya latistoma* Fritsch—Romer, 1947: pp. 104–106, fig. 20 *non* Fritsch, 1885.

*Gaudrya latistoma* Fritsch—Milner, 1980: p. 453 *non* Fritsch, 1885.

*Capetus palustris* Steen—Sequeira & Milner, 1993: figs. 1–10, 12C; pls. 1–3.

*Baphetes bohemicus* (Fritsch)—Štamberg & Zajíc, 2008: fig. 248.

*Loxomma bohemicum* Fritsch (*nomen dubium*)—Milner et al., 2009: p. 320, fig. 1A.

‘*Loxomma*’ *bohemicum* Fritsch—Clack & Milner, 2015: p. 61.

*Holotype*: BMNH R4706, an imprint of a skull table and cheek (Sequeira & Milner, 1993).

*Locality and horizon*: Nýřany, 13 km south‐west of Pilsen, West Bohemia, Czech Republic. Main Nýřany Coal Seam, Nýřany Member, Kladno Formation in Pilsen Basin; Middle Pennsylvanian (Moscovian: Asturian).

*Diagnosis*: For the diagnosis see Sequeira and Milner (1993).

#### 2.3.4 The type material of *Loxomma bohemicum* Fritsch, 1885

**FIGURE 6:**
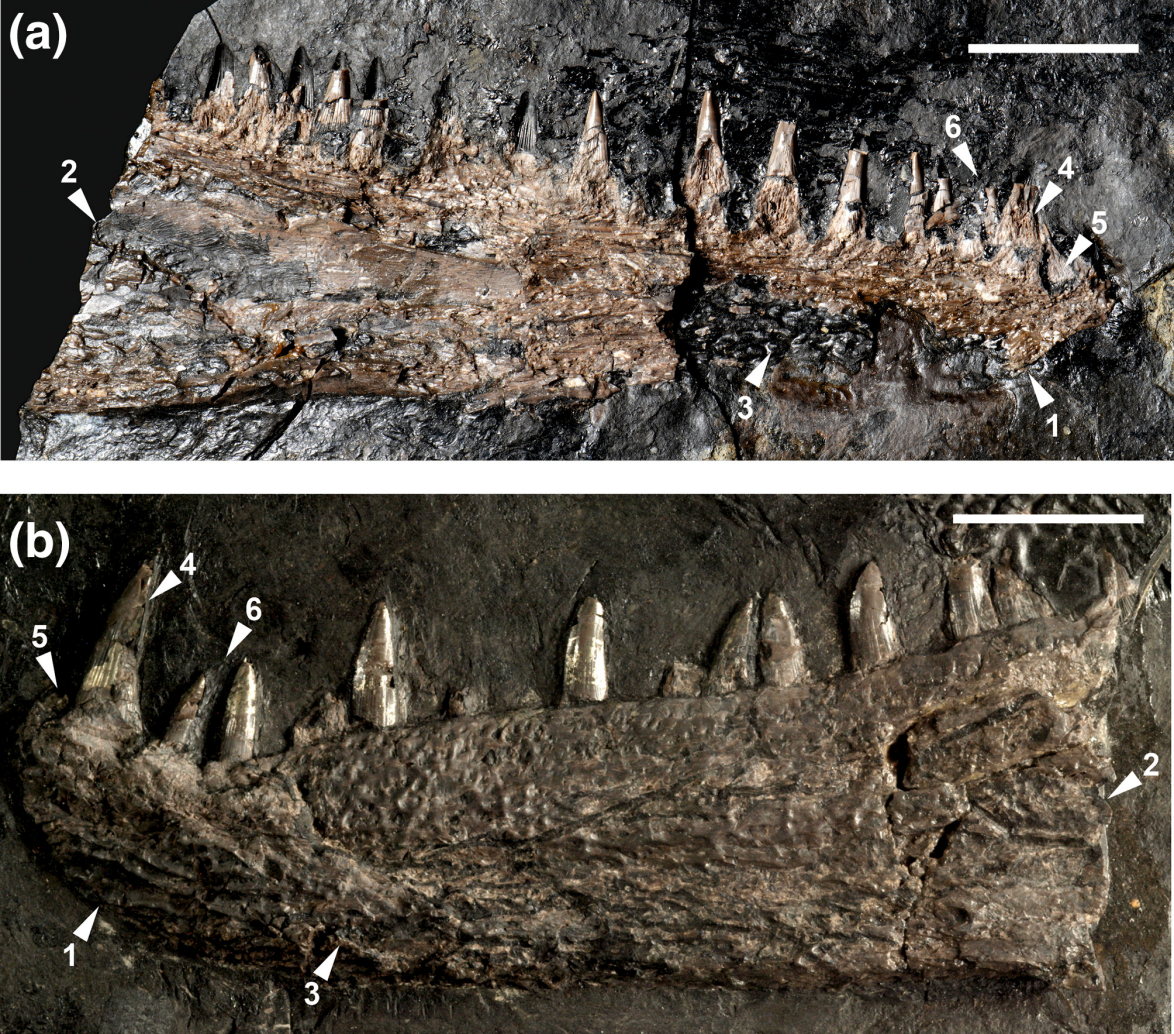
The comparison of *Loxomma bohemicum* with the specimen referred to *Capetus palustris*. (a) The type specimen of *Loxomma bohemicum*, NMP M535, Middle Pennsylvanian, Nýřany, Czech Republic, isolated left dentary from medial view. (b) The specimen referred to *Capetus palustris*, NHMW‐Geo‐1898/0010/0051a, Middle Pennsylvanian, Nýřany, Czech Republic, left dentary from lateral view. The shared characteristics of both specimens are marked by arrowheads (1–6) and explained in the text. Scale bars = 20 mm.

The specimen NMP M535 represents a poorly preserved anterior portion of the left dentary in medial view, having badly disrupted superficial bone structure, and measuring 124 mm in length (Figure 6a). The orientation of the jaw fragment indicates it represents the specimen originally from Bayer's private collection in Pilsen rather than the one figured by Fritsch (1885, pl. 58, fig. 3) from the former German Charles‐Ferdinand University in Prague. However, Fritsch (1885) noted the specimen figured by him represents the left lower jaw exposed from the external (lateral) view. Based on the nature of the specimen examined here, as well as the original Fritsch figure, we conclude that the material has been split into two pieces (part and counterpart) in such a way that the disrupted internal (medial) structure of the bone is preserved on both parts of the specimen. However, a small portion of the bone's lateral surface remains preserved in the anteroventral region of NMP M535.

The anterior‐most portion of the jaw ramus is relatively narrow, blunt, and curved dorsally, but its width gradually increases posteriorly to the point the bone is broken off. The ventral margin is nearly straight anteriorly, whereas the dorsal edge slopes at a low angle. The anteroventral part of the bone is broken off, showing the imprint of the lateral dermal sculpture consisting of coarse pits and ridges. No details of the medial surface can be described due to the disrupted bone structure. There are 17–18 teeth preserved in the jaw ramus of the specimen, but, owing to its incomplete nature, the total tooth number is difficult to determine. The teeth are upright and appear to be roughly similar in size, although a distinct variation in proportions can be seen in some parts of the dentition as well. The anterior‐most tooth is slender and very small, reaching less than half the height of the largest teeth. It appears to be rotated 90° and shows an apically compressed tip bearing a sharp cutting edge. The subsequent tooth has a wider base than most of the other teeth, but the apical portion is not preserved to infer its original length. It was likely larger than the surrounding dentition in the anterior part of the mandible, forming a prominent dentary fang, but it probably did not exceed the length of the teeth placed at the jaw mid‐length. Behind this dentary fang, about three or four smaller teeth are preserved in a variably broken state, followed by four larger teeth at the mid‐length of the jaw. The dentition in this region is relatively widely spaced, and several teeth show nearly complete apical parts that are smooth, laterally compressed, and possess prominent carinae. Behind the mid‐length of the preserved jaw fragment, the teeth are smaller, closely spaced, and constant in size. All teeth have fine longitudinal grooves at the base.

*Remarks*: *Loxomma bohemicum* was first described and figured by Fritsch (1885, pl. 58, figs. 3–9) based on an isolated lower jaw fragment in part and counterpart. This material is taxonomically important, as it represents the historically first putative baphetid species recognized in the late Carboniferous of the Czech Republic. Consequently, its affinity has implications for the taxonomy of the Nýřany baphetids. Steen (1938) considered the material to be indeterminable, while Milner et al. (2009) noted the specimen could not be located, assuming it is lost. They pointed to its large dimensions and general resemblance with *Capetus palustris*, concluding it represents a *nomen dubium*. A part of this supposedly lost type material was recently rediscovered in the collections of the National Museum in Prague, enabling the reassessment of its taxonomic position.

Fritsch (1885) referred the specimen to the genus *Loxomma* on the basis of a sharp cutting edge present in the marginal teeth of the jaw fragment, resembling the material from the Newsham (Northumberland, United Kingdom) ascribed by Embleton and Atthey (1874) to *Loxomma allmanni*, which is now attributed to *Megalocephalus pachycephalus* (Beaumont, 1977). The relatively narrow anterior portion of the jaw ramus, as well as the arrangement and form of the teeth, indeed generally resemble baphetids such as *M. pachycephalus*, although the details of the mandible also show some differences. In contrast to *M. pachycephalus*, *“Loxomma” lintonensis* and *Baphetes orientalis* (including ÚGV PAL00220), the dentary of *Loxomma bohemicum* is greatly expanded posteriorly rather than being relatively uniform in width throughout its length. It also appears to lack the thin sheet of bone covering the bases of the teeth, present in *M. pachycephalus* and ÚGV PAL00220. The very small anterior‐most tooth is absent in *Baphetes orientalis* and *“Loxomma” lintonensis*, although it is present in *M. pachycephalus* (Ahlberg & Clack, 1998; Beaumont, 1977). However, the dentition of *Loxomma bohemicum* does not show the distinctive enlargement of the teeth in the same manner as seen in *M. pachycephalus*, with differences in tooth size being rather small, especially at the jaw mid‐length (Ahlberg & Clack, 1998; Beaumont, 1977; Embleton & Atthey, 1874). These limited comparisons indicate that *Loxomma bohemicum* cannot be confidently ascribed to any baphetid known at the present time.

Milner et al. (2009) suggested that the jaw fragment of *Loxomma bohemicum* could belong to the large temnospondyl amphibian *Capetus palustris* based on its immense proportions, and they pointed to its morphological resemblance with the specimen NHMW‐Geo‐1898/0010/0051a (Sequeira & Milner, 1993, fig. 8B). Here we compare the morphology and dimensions of a well‐preserved lower jaw fragment, referred to *Capetus palustris* (NHMW‐Geo‐1898/0010/0051a), with that of *Loxomma bohemicum* to elucidate whether they might belong to the same species (Figure 6a,b). Both specimens have similar proportions and match in the preserved part, enabling a good comparison (Table 1). Unlike Sequeira and Milner (1993), we interpret the anterior‐most portion of the *Capetus* mandible to be completely preserved. *Loxomma bohemicum* shares with *Capetus palustris* the following characteristics: (1) the slender, rounded, and dorsally curved anterior part of the dentary at the level of the mandibular symphysis, bearing a concave dorsal margin; (2) the dentary ramus gradually widens posteriorly to become greatly expanded dorsoventrally; (3) the anteroventral part of the dentary bears a dermal sculpture formed by rugose pits and ridges in lateral view; (4) a large dentary fang is present in the symphysial region; (5) a very small anterior‐most tooth present in front of the dentary fang (a tooth base is seen in the jaw referred to *C. palustris*); (6) several smaller teeth present immediately posteriorly to the dentary fang, the smallest ones reaching about half its length (Figure 6a,b). Although the marginal dentition is broken and incomplete in both specimens, its general form and arrangement appear to be nearly identical. The teeth at the mid‐length of the jaw ramus are all incomplete in the *C. palustris* lower jaw, thus it is not possible to compare them with the well‐preserved dentition of *Loxomma bohemicum*. However, the posterior teeth appear to be small and densely spaced, as is the case with the latter. Moreover, the teeth are upright and bear a laterally compressed, smooth apical part with a distinct cutting edge in *C. palustris*, thus well conforming in morphology to that seen in *Loxomma bohemicum*. Finally, the base of the teeth shows fine longitudinal grooves extending to the mid‐length of the tooth crown in both specimens. Based on the morphological comparisons and measurements, we find only very subtle differences, mostly restricted to the dimensions, between the type specimen of *Loxomma bohemicum* and the jaw fragment referred to *Capetus palustris*. These might well be explained by the intraspecific variation. Thus, we formally propose here that the type lower jaw of *Loxomma bohemicum* represents a poorly preserved specimen of *Capetus palustris* and may be excluded from the hypodigm of *Baphetes orientalis*, as well as baphetids in general.

**TABLE 1:** Measurements in the type dentary of *Loxomma bohemicum* compared to the specimen referred to *Capetus palustris* (NHMW‐Geo‐1898/0010/0051a).

Measured parameter	*Loxomma bohemicum*	*Capetus palustris*
Total length of preserved fragment (mm)	124	113
Width at the anterior dentary fang level (mm)	13	18
Width at the jaw mid‐length (mm)	23	29
Width at the posterior‐most jaw level (mm)	36	39
Length of the anterior‐most tooth (mm)	7	2^a^
Width of the anterior‐most tooth at the base (mm)	3	3
Length of the anterior fang (mm)	11^a^	19
Width of the anterior fang at the base (mm)	6	8
Length of the tooth at the jaw mid‐length (mm)	13	11^a^
Width of the tooth base at the jaw mid‐length (mm)	6.5	5
Estimated number of teeth in the preserved part	21–22	20–21

a The measured structure is incomplete.

### 2.4 Phylogenetic analysis

In order to resolve the internal relationships of the Baphetoidea, we modified and expanded the original data matrix of Milner et al. (2009), which consists of 47 characters and 16 taxa (Appendix S1). All characters were treated as equally weighted and unordered; multistate characters were interpreted as polymorphisms. *Acanthostega*, *Crassigyrinus*, and *Greererpeton* were defined as outgroup taxa. The juvenile and adult specimens of *Baphetes orientalis* were treated as separate OTUs (operational taxonomic units) for testing their conspecificity. The taxonomic concept of *Megalocephalus lineolatus* and *Loxomma lintonensis* as separate taxa (Beaumont, 1977; Clack & Milner, 2015; Milner et al., 2009) is followed here (*contra* Hook & Baird, 1986: p. 181). *Baphetes planiceps* was, after the initial analysis, excluded from the taxon sample due to its incompleteness affecting the tree resolution.

**FIGURE 7:**
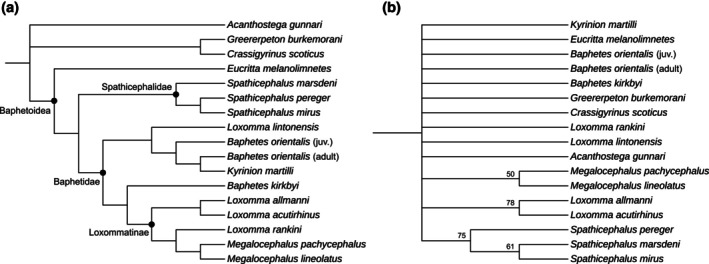
The phylogenetic analysis of the Baphetoidea. (a) The single most‐parsimonious tree with the length of 129 steps. (b) The bootstrap analysis showing support for each node (clades recovered in less than 50% of cases were collapsed into polytomy).

The analysis resulted in a single most parsimonious tree (MPT) with a length of 129 steps (Figure 7a; Consistency Index [CI] = 0.543; Retention Index [RI] = 0.556; Homoplasy Index [HI] = 0.465). The resulting topology recovered monophyletic Baphetoidea with *Eucritta melanolimnetes* as the basal‐most member of the clade. All three species of *Spathicephalus* (*S. marsdeni*, *S. mirus*, and *S. pereger*) form the sister–clade (Spathicephalidae) relationships to the Baphetidae. *“Loxomma” lintonensis*, *Baphetes orientalis*, and *Kyrinion martilli* form a basal clade within baphetids as successively more derived taxa. We did not find sister–taxon relationships of the juvenile and adult specimens of *Baphetes orientalis*, with the latter placed close to *Kyrinion martilli* in the present analysis. The second major clade recovered within the Baphetidae consists of *Baphetes kirkbyi*, followed by the small clade formed by *Loxomma acutirhinus* and *L. allmanni* in sister–group relationships to *Loxomma rankini* and *Megalocephalus lineolatus* + *M. pachycephalus*. The bootstrap support for most of the clades is low (<50%), resulting in a collapse into a large basal polytomy, with few clades reasonably well supported (Figure 7b).

## 3 DISCUSSION

### 3.1 Interrelationships of the Baphetoidea

The results of our phylogenetic analysis show a general resemblance to the topology recovered by Milner et al. (2009), although some aspects of the baphetoid phylogeny appear to be less consistent with the previous phylogenetic hypotheses (Figure 7a). *Eucritta melanolimnetes* was originally considered to represent the basal‐most baphetoid (Clack, 1998, 2001), a result corroborated by some phylogenetic hypotheses of early tetrapod relationships (Pardo et al., 2017; Ruta & Clack, 2006; Ruta & Coates, 2007; Smithson et al., 2024), while others recovered it in a more derived phylogenetic position close to temnospondyls or stem‐amniotes (Clack et al., 2016, 2019; Ruta et al., 2003). Although we found *E. melanolimnetes* in the basal‐most position relative to all other baphetoids, it must be emphasized that the dataset used in the present analysis is not designated to test the phylogenetic position of this taxon among other basal tetrapods, thus it cannot contribute to this problem. We included all three species of the genus *Spathicepahlus* in the phylogenetic analysis for the first time to test the monophyly of the Spathicephalidae (Beaumont & Smithson, 1998) relative to other baphetoids, as well as their internal relationships. This clade is recovered here as one of the most robustly supported (Figure 7b), and, in accordance with previous hypotheses, is placed as a basal sister group to baphetids (Beaumont & Smithson, 1998; Milner et al., 2009), with *S. marsdeni* and *S. pereger* variably found in the basal‐most position of the clade. Baphetidae is divided here into two subclades, the content of which, however, differs from the results of Milner et al. (2009), most importantly in the position of *Baphetes kirkbyi*, *“Loxomma” lintonensis* and *Kyrinion martilli*. *B. kirkbyi* is found to be more closely related to the *Loxomma–Megalocephalus* clade (Loxommatinae sensu Milner et al., 2009), rather than being associated with *Baphetes orientalis* to form a monophyletic Baphetinae (sensu Milner et al., 2009). This result is not entirely surprising considering one of the formerly obtained topologies, where *B. kirkbyi* formed an unresolved polytomy with *B. orientalis* and the remaining baphetids (Milner et al., 2009, fig. 6B), indicating that the genus *Baphetes*, and, consequently, the subfamily Baphetinae, might be polyphyletic. Our results do not support the close relationships of *“Loxomma” lintonensis* with either *Loxomma allmanni* or any other species of the genus *Loxomma*, as previously suggested by Milner et al. (2009), who, based on the results of their phylogenetic analysis, transferred the species into the genus *Loxomma*. In fact, *L. allmanni* and *L. acutirhinus* form one of the few well‐supported clades (78% bootstrap support; Figure 7b) in the present analysis, whereas *“Loxomma” lintonensis* is returned to a position more closely related to *Baphetes orientalis* than to other *Loxomma* species (Figure 7a). In contrast to the hypothesis of Milner et al. (2009), *Kyrinion martilli* is recovered here as forming a clade with *“L.” lintonensis* and *B. orientalis*, rather than being a highly derived loxommatine close to *Megalocephalus pachycephalus*, although the bootstrap support of this clade is low. The adult specimen of *Baphetes orientalis* is found to be in sister–taxon relationships with *Kyrinion martilli*, instead of being closely related to the juvenile individual (*contra* Milner et al., 2009). We do not consider this result to be particularly well‐supported to challenge the taxonomic placement of both specimens in the same species (as different ontogenetic stages; Milner et al., 2009), and it might well be affected by ontogenetic variability or the unstable phylogenetic position of *Kyrinion*. Finally, our results agree with the previous hypothesis that the genus *Loxomma* (including *L. allmanni*, *L. acutirhinus*, and *L. rankini*) is paraphyletic with respect to *Megalocephalus* (Clack & Milner, 2015; Milner et al., 2009), although *M. lineolatus* and *M. pachycephalus* form a clade in the present analysis.

In general, the baphetids (especially *Baphetes* and *Loxomma*) display a high degree of plesiomorphic features in the skull, making it difficult to establish clear synapomorphies for them. However, one important character in baphetid phylogeny appears to be the intertemporal bone, which undergoes an interesting evolution towards the gradual reduction. Indeed, it is a fairly prominent bone in the dermal skull table of *Baphetes orientalis*, *B. kirkbyi*, and *“Loxomma” lintonensis* (the state is unknown in *Kyrinion*), but its size is greatly reduced in *Loxomma acutirhinus*, *L. allmanni*, and *L. rankini*, until it finally disappears in *Megalocephalus lineolatus* and *M. pachycephalus*. According to the topology obtained here, the independent loss of the intertemporal bone occurred at least twice in the baphetoid evolution: the first time in spathicephalids and the second time in loxommatines. A similarly continuous character transition could possibly be traced also in the snout morphology and premaxillary tooth number of baphetids. The snout is very broad in adult specimens of *Baphetes orientalis*, *B. kirkbyi*, and *B. planiceps*, but shows some degree of tapering in *Loxomma acutirhinus*, followed by the even more tapered condition in *Megalocephalus*. The premaxilla of *Baphetes orientalis* and *B. kirkbyi* bears 10–11 teeth, but in *Loxomma acutirhinus* and *Megalocephalus pachycephalus*, its number is reduced to eight. This stepwise character evolution could further support the hypothesis on the paraphyletic nature of the *Loxomma*‐grade and challenge the monophyletic status of the genus *Baphetes* relative to Loxommatinae.

The results of our phylogenetic analysis underscore some problems of the current baphetoid systematic and taxonomy proposed by the previous authors (Beaumont, 1977; Clack & Milner, 2015; Milner et al., 2009), indicating lesser phylogenetic stability within the clade than formerly assumed. The most problematic part of the baphetoid phylogeny appears to be the current taxonomic conception of the genus *Baphetes* affecting the monophyly of the Baphetinae, which is reflected by the unstable position of *Baphetes kirkbyi* and *Kyrinion martilli*. In fact, the monogeneric Baphetinae can only be characterized on the basis of several symplesiomorphic features, such as a broad snout, premaxilla bearing 10–11 teeth, large intertemporal bone, and nasals extending anterior to the naris (see also Clack & Milner, 2015). As a result, the highly conservative skull morphology present in the members of the group warrants detailed reassessment of the key characteristics to determine whether various *Baphetes* species share any apomorphic characteristics to form a clade, or rather represent a polyphyletic assemblage. Likewise, the original taxonomic conception of the genus *Loxomma* (Beaumont, 1977) is in need of thorough revision, since at least *L. rankini* appears to be more closely related to *Megalocephalus* than to other *Loxomma* species (see Clack & Milner, 2015). The problem related to the taxonomic status of *“Loxomma” lintonensis* is more thoroughly discussed below.

### 3.2 Taxonomic identity of *Loxomma lintonensis* (Beaumont, 1977)

A small dermal skull roof with palate and associated mandibles from the Middle Pennsylvanian of the Linton locality (Cope, 1875b: pl. 37, fig. 3; Romer, 1930: fig. 20) was described by Beaumont (1977) as a new species, *Baphetes lintonensis* Beaumont, 1977. Hook and Baird (1986) argued that the type specimen of another Linton baphetid, *Megalocephalus lineolatus* (Cope, 1877), is assignable to the genus *Baphetes*, thus establishing a new combination, *Baphetes lineolatus* (Cope, 1877), and also referred to it the single specimen of *Baphetes lintonensis* Beaumont, 1977, which became its junior synonym. However, no morphological evidence was put forward to corroborate this taxonomic conclusion, and it was not followed by subsequent authors (Clack & Milner, 2015; Milner et al., 2009). Recently, Milner et al. (2009) argued for the taxonomic attribution of *Baphetes lintonensis* in the genus *Loxomma* based on the results of their phylogenetic analysis and the presence of the following characters: (1) a broad snout region that may change to a *Loxomma*‐type morphology during ontogeny; (2) the antorbital vacuities no larger than the orbits; and (3) the presence of maxilla‐quadratojugal contact. The type and only specimen of *“Loxomma” lintonensis* is clearly a juvenile individual (Beaumont, 1977), thus it is reasonable to compare its features with *Baphetes orientalis*, the single baphetid species for which ontogenetic changes can be evaluated. The relatively broad snout region of *“L.” lintonensis* is shared with NHMW‐Geo‐1898/0010/0042 (juvenile specimen of *B. orientalis*), with the width/length ratio reaching between 1.6 and 1.8 in both specimens (measured in front of the antorbital vacuities and from the anterior border of the antorbital fenestra to the anterior margin of nasals). Thus, the snout proportions of the juvenile *“L.” lintonensis* may equally result in a broad snout morphology seen in the adult specimens of *Baphetes*, and, due to the lack of ontogenetic data in *Loxomma*, cannot be used to distinguish between the genera in question. The exact boundary between the orbit and the antorbital fenestra is difficult to establish and subsequently compare in various baphetid species. In order to facilitate the comparison, the extent of the antorbital fenestra is defined here as delimited by the medially bulged jugal (or jugal‐postorbital suture in the taxa lacking this feature), laterally bulged prefrontal, and the lacrimal. The dimensions of the antorbital vacuities as defined here are larger than the orbits in *“L.” lintonensis*, as well as both specimens of *Baphetes orientalis*, *B. kirkbyi*, and *Loxomma allmanni*, thus this feature seems to be widespread among the known baphetid species. Finally, the maxilla‐quadratojugal contact present in *“L.” lintonensis* probably represents an ontogenetically influenced character, as already noted by Beaumont (1977), and confirmed by the different character states present in the juvenile and adult specimens of *Baphetes orientalis* (see below). Therefore, none of the characters formerly used to support the attribution of the Linton specimen to the genus *Loxomma* appears to be justified at the present time. The two morphological features that distinguish *Loxomma*‐grade from all the *Baphetes* species, and seem not to be ontogenetically influenced, are (1) the presence of relatively small intertemporal (<50% supratemporal length; see also Clack & Milner, 2015) and (2) the nasals not extending anterior to the external naris (Beaumont, 1977), both characters being absent in *“L.” lintonensis*. Thus, the results of our phylogenetic analysis, as well as the comparison of the relevant characters, call into question the recent taxonomic placement of the Linton species in the genus *Loxomma*, which is considered here to be premature and insufficiently supported by the morphological data. However, the attribution of this species to *Baphetes* is currently supported only by a few primitive characteristics mostly shared with other stem‐tetrapods (e.g., broad and rounded snout, large intertemporal, anterior extension of nasal). This underlines the more complex problematic of the taxonomic concept of the genus, which shows almost no apomorphic features with respect to *Loxomma* and may ultimately turn out to be heterogeneous in nature.

Concerning the species‐level taxonomy, Beaumont (1977) based the diagnosis of the species on a single character (i.e., maxilla–jugal contact), which is ontogenetically influenced, whereas Clack and Milner (2015) found no diagnostic features to differentiate the species. *“Loxomma” lintonensis* can be distinguished from the similarly sized juvenile specimen of *Baphetes orientalis* by the following characteristics (based on Beaumont, 1977): (1) the skull table dermal sculpture composed of deep pits; (2) lateral constriction of orbit formed by bulging jugal and postorbital; (3) the lacrimal process of jugal extends posterior to the anterior antorbital fenestra margin; (4) the postfrontal process of postorbital relatively wide and rounded; (5) the postorbital lacks a narrow, quadrangular lateral process; (6) the postorbital‐supratemporal suture point‐like; (7) the cheek region extends far posterior to the skull table; (8) two anterior dentary fangs present at the same time; and (9) the absence of an elongate and slender anterior process of angular. The short, point‐like contact between the postorbital and supratemporal is identified and proposed here as the only autapomorphic character distinguishing *“L.” lintonensis* from all other known baphetid species, although the relevant part of the skull is missing in *Megalocephalus lineolatus*. Pending future revisions of this and other relevant baphetids, the current generic status of the Linton specimen is retained here as provisional and given with quotation marks, although several lines of evidence indicate a *Baphetes*‐like affinity of the species. Similarly, a possible division of the *Megalocephalus lineolatus* hypodigm and its putative association with *“Loxomma” lintonensis* (see Hook & Baird, 1986) needs to be tested in order to elucidate the specific taxonomic position of the latter.

### 3.3 Ontogenetic changes in *Baphetes orientalis*

**FIGURE 8:**
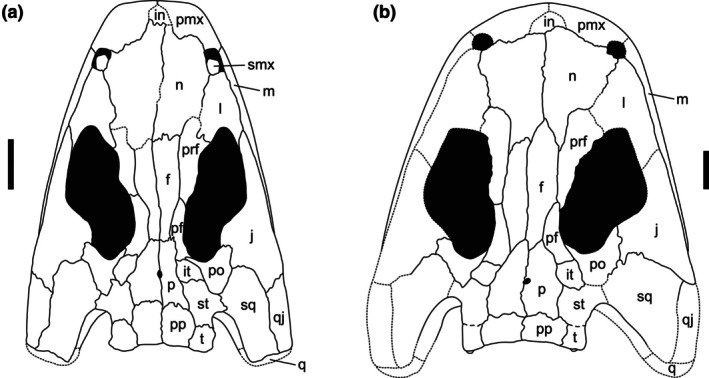
The interpretative skull reconstructions of the juvenile (a) and the possible adult (b) specimens of *Baphetes orientalis*, Middle Pennsylvanian, Nýřany, Czech Republic, from the dorsal view. Scale bars = 10 mm (a), 20 mm (b).

The baphetoid ontogeny is currently very poorly understood based on the morphological data, since no larval stages have been so far discovered and only two juvenile individuals referred to *“Loxomma” lintonensis* and *Baphetes orientalis* are known from the Moscovian of Linton and Nýřany, respectively (Beaumont, 1977; Milner et al., 2009; Romer, 1930). Furthermore, it is difficult to infer their possible mode of development from a phylogenetic framework, since the group has been historically placed either to the close relationships with temnospondyls (Clack, 1998, 2001, 2003; Gauthier et al., 1989; Romer, 1947) or more plausibly as stem‐tetrapods close to the crown group (Clack et al., 2016, 2019; Gardiner, 1983; Laurin & Reisz, 1997; Marjanović & Laurin, 2019; Pardo et al., 2017; Ruta et al., 2003; Ruta & Clack, 2006; Ruta & Coates, 2007; Smithson et al., 2024), both of which are expected to show different patterns in the ontogenetic development. In baphetids, growth changes can currently only be documented in *Baphetes orientalis*, based on the different character states present in the very small (about 70 mm) and much larger (180 mm) skull (Figure 8), first documented by Milner et al. (2009). Additionally, an isolated right dentary (ÚGV PAL00220) with the length of 65 mm is referred here to this species as well (Figures 4 and 5). Based on the comparison with other baphetids, in which the dentary comprises about 70%–80% of the mandibular length (Beaumont, 1977), it would pertain to the individual with a skull length of approximately 80–90 mm, thus being slightly larger than NHMW‐Geo‐1898/0010/0042. Therefore, this small sample currently represents the most complete ontogenetic sequence of any baphetid reported to date. The morphological revision of all Nýřany baphetid material carried out here results in some novel ontogenetic data, which complement and refine those reported by previous authors (Milner et al., 2009).

NHMW‐Geo‐1898/0010/0042 represents a juvenile individual based on its small total skull length and the character of the dermal sculpture, which consists of radiating ridges and grooves on most of the dorsal skull bones (Bystrow, 1935). Although the holotype shows the reticulate sculpture on the majority of the dermal skull roof, indicating a possible adult stage, the coarsely radiating pattern can be seen in the nasal, lacrimal, jugal, and squamosal, suggesting the individual might still have been in the process of growth (Figures 1c and 2c). The dentary of the smallest individual (NHMW‐Geo‐1898/0010/0042) shows fine longitudinal striations on the lateral surface, whereas the larger isolated dentary (ÚGV PAL00220) exhibits only a few ridges and grooves (Figures 2c and 4c). This pattern of lateral dermal sculpture is consistent with the juvenile *“Loxomma” lintonensis* but differs from the reticulate ornamentation reported in the large specimen of *Baphetes* cf. *kirkbyi* (Milner & Lindsay, 1998) and *Megalocephalus pachycephalus* (Beaumont, 1977). The conspicuous pits around the symphysial region, present in ÚGV PAL00220, are, however, not related to the dermal sculpture but represent the nutrient foramina (Figure 5b) similar to those figured in *“Loxomma” lintonensis* (Beaumont, 1977, fig. 24B,E). The ossification of the dermal skull roof is already completed in the juvenile individual, and the specimen generally resembles the adult form, indicating gradual anatomical transformation plesiomorphic for the stem‐tetrapods, rather than the drastic developmental changes accumulated over a short period of time and currently restricted to several temnospondyl lineages (Schoch & Witzmann, 2024). The morphology of the snout appears to undergo some developmental changes related to the proportions of the lacrimal and the anterior process of the jugal, discussed in more detail by Milner et al. (2009). The latter structure is more elongated and narrower in the juvenile specimen, reaching the anterior margin of the antorbital fenestra, whereas it is shortened and robust in the adult individual (Figure 8). Interestingly, this anterior extension of the jugal appears to be absent in *“Loxomma” lintonensis*; thus, it may indicate differences in ontogenetic development between the two species. Another character with possible ontogenetic significance is the maxilla‐quadratojugal contact, which is present in the juvenile specimen of *B. orientalis* (NHMW‐Geo‐1898/0010/0042) and was tentatively used by Beaumont (1977) to diagnose *“Loxomma” lintonensis*. Milner et al. (2009) scored the character state as unknown in the holotype of *Baphetes orientalis*, although Beaumont (1977) noted that the displaced right maxilla is complete posteriorly, based on its length consistent with the left element. Besides this, the posterior‐most part of the right maxilla is slender and pointed, and the dentition appears to terminate some distance before its end (Figure 3a,b), indicating it is indeed complete or near complete posteriorly. As a result, the lateral extension of the jugal excludes the maxilla from the quadratojugal contact in the adult *Baphetes orientalis* specimen, as it also does in *Baphetes kirkbyi* (Beaumont, 1977). A conspicuous feature of the juvenile *Baphetes orientalis* is the lateral constriction of the orbit formed exclusively by the bulging jugal. Milner et al. (2009) considered this character to be diagnostic of the species, pointing to its subtle development in the holotype depicted by Beaumont (1977). However, the reassessment of the type specimen showed that both the jugal and the postorbital contribute to the bulged region; thus, this peculiar character is to be likely ontogenetically variable and specific to the ontogeny of *Baphetes orientalis* (Figure 8). We cannot confirm ontogenetic variability related to the extension of the supratemporal along the anterior margin of the temporal notch in *Baphetes orientalis*, proposed by Milner et al. (2009). While the supratemporal‐squamosal suture is well apparent in NHMW‐Geo‐1898/0010/0042 and the bone appears to significantly participate in the anterior margin of the otic notch, the exact boundary of the squamosal and the supratemporal cannot be reliably discerned in the holotype, preventing an evaluation of its condition. If the morphology of the supratemporal conforms to that of other baphetids, then no clear difference between juvenile and adult specimens of this species can be observed. The ontogenetic change in the shape of the postparietal was accurately described by Milner et al. (2009). The tabular horns are absent in NHMW‐Geo‐1898/0010/0042, but they are subtly developed in the holotype of *Baphetes orientalis*, although they are much smaller than those figured in *Baphetes kirkbyi*, *Loxomma acutirhinus*, and *Megalocephalus pachycephalus* (Beaumont, 1977). The condition present in juvenile *“Loxomma” lintonensis* is unclear. These structures might have increased with the skull growth during ontogeny. Milner et al. (2009) suggested that the lateral line canals, developed in the postorbital region of *“Loxomma” lintonensis* and both specimens of *Baphetes orientalis*, may be an ontogenetic feature absent in large baphetid skulls. Based on our re‐evaluation of the *Baphetes orientalis* anatomy, we found no conclusive evidence on the presence of the lateral line sulci on the postorbital and postfrontal in either specimen (Figures 1c and 2c). In accordance with *Baphetes* cf. *kirkbyi* (Milner & Lindsay, 1998), the lateral line canal is placed on the jugal in both individuals, although it varies in the exact position on the bone.

### 3.4 Taxonomy, diversity and palaeoecology of Nýřany baphetids

The morphological revision of all Nýřany specimens historically referred to baphetids, as well as the description of the new material, enables a re‐evaluation of the baphetoid taxonomy and diversity at the locality. The type lower jaw fragment, described as *Loxomma bohemicum* Fritsch, 1885, most likely belongs to a large temnospondyl amphibian, *Capetus palustris* Steen, 1938, and should no longer be considered in baphetoid taxonomy. As a result, *Baphetes orientalis* Milner et al., 2009 is retained as the valid name for the Nýřany species (for the discussion on the taxonomic nomenclature, see Milner et al., 2009), with NMP M1388 designated as the holotype. NHMW‐Geo‐1898/0010/0042 represents a small skull with two mandibles and a partial pectoral girdle which can be referred to *Baphetes orientalis* on the basis of a sharply pointed postfrontal process of postorbital and slightly elongate, rectangular lateral process of the bone. Finally, the isolated right dentary (ÚGV PAL00220) resembles that of NHMW‐Geo‐1898/0010/0042 and is tentatively attributed here to *Baphetes orientalis* as well. Thus, a single baphetid species is currently known from the Middle Pennsylvanian (Moscovian) of the Czech Republic, whereas the contemporaneous coal‐bearing fauna at the Linton locality contains at least two baphetid species, *“Loxomma” lintonensis* and the more derived *Megalocephalus lineolatus* (Clack & Milner, 2015). All three species currently represent the latest stratigraphic records of the clade.

Baphetids exhibit a number of characteristics thought to represent adaptations for the piscivorous aquatic lifestyle, such as a well‐developed lateral line system, a large antorbital fenestra possibly accommodating a massive pterygoideus musculature that allowed rapid jaw closure against water pressure, sharp lanceolate teeth, and stout palatal and dentary tusks for catching fish prey (Beaumont, 1977; Romer, 1947; Taylor, 1987). In contrast, the more basal spathicephalids were likely specialized aquatic filter‐feeding forms, as indicated by their numerous small, densely arranged blunt teeth (Beaumont & Smithson, 1998; Smithson et al., 2017). Baphetids are frequently found in the coal‐bearing lake deposits with abundant aquatic vertebrate fauna (Boyd, 1984; Hook & Baird, 1986; Milner, 1980), and *Baphetes orientalis* might have fed on some large‐bodied actinopterygian fishes recently reported from the Nýřany assemblage (Barták et al., 2024), as well as dipnoans and xenacanthiform sharks known to occur at the locality (Fritsch, 1889; Heidtke, 1998; Schneider & Zajíc, 1994; Soler‐Gijón, 2004). Barták et al. (2024) suggested that, based on its rare occurrence in coal deposits of Nýřany, *Baphetes orientalis* likely preferred the braided river system flowing through the alluvial plain of the Nýřany Member, with occasional excursions to a relatively shallow lake. This may be supported by the presence of specimens with complete or near complete articulated skulls (NMP M1388, NHMW‐Geo‐1898/0010/0042) showing only minor effects of transportation, as well as isolated elements (ÚGV PAL00220) possibly transported to the place of deposition from a greater distance. The presence of various ontogenetic stages in the same depositional environment may indicate that no significant ecological shift between juveniles and adults took place during the ontogeny of *Baphetes orientalis*, although the sample is too small to make unambiguous conclusions.

## 4 CONCLUSIONS

The morphological revision of all available material previously attributed to baphetids from the Middle Pennsylvanian (Moscovian: Asturian) of the Nýřany locality (Czech Republic), including one newly described specimen, was performed. A part of the supposedly lost type material of *Loxomma bohemicum* has been discovered in the collections of the National Museum in Prague and redescribed for the first time, showing that based on its morphology and dimensions it most likely belonged to a poorly preserved specimen of the large temnospondyl amphibian *Capetus palustris*. All other baphetid material can be referred to various growth stages of a single species, *Baphetes orientalis*, which, despite being largely incomplete, currently represents the most informative sample on baphetid ontogeny. The species shows several morphologically variable characters in the skull of juvenile and adult specimens, which are interpreted as related to the unique ontogenetic development of baphetids. The most comprehensive phylogenetic analysis of the Baphetoidea to date has been carried out, the results of which indicate that the genus *Baphetes* may be polyphyletic, whereas *Loxomma* is paraphyletic with respect to *Megalocephalus*. *“Loxomma” lintonensis* is returned to a position close to *Baphetes orientalis*, and its previous taxonomic attribution to the genus *Loxomma* is discussed and shown to be doubtful based on available morphological data. *Baphetes orientalis* was an aquatic piscivorous predator possibly feeding on large actinopterygian fishes, dipnoans and xenacanthiform sharks, and predominantly living in the river system flowing through the alluvial plain of the Pilsen Basin.

## AUTHOR CONTRIBUTIONS

**Pavel Barták:** Conceptualization; investigation; writing – original draft; methodology; visualization; software; formal analysis. **Martin Ivanov:** Conceptualization; funding acquisition; writing – review and editing; supervision. **Boris Ekrt:** Methodology; visualization; resources.

## Supporting information


**APPENDIX S1:** Supporting information.
