# Supplementary material for: The revision of baphetids from the Middle Pennsylvanian of the Czech Republic: Morphology, ontogeny, palaeoecology, and the reassessment of the phylogeny of Baphetoidea
Source: Anat Rec (Hoboken). 2025 Sep 19;309(7):1766–87. doi: 10.1002/ar.70054 (PMC13251752; doi:10.1002/ar.70054)
Supplement: Supplementary file 1 — APPENDIX S1: Supporting information. [file AR-309-1766-s001.docx]

SUPPLEMENTARY FILE

**The revision of baphetids from the Middle Pennsylvanian of the Czech Republic: morphology, ontogeny, palaeoecology, and the reassessment of the phylogeny of Baphetoidea**

PAVEL BARTÁK^1,2*^, MARTIN IVANOV^1^ and Boris Ekrt^3^

^1^ Department of Geological Sciences, Faculty of Science, Masaryk University, Kotlářská 267/2 611 37 Brno, Czech Republic

^2^Ostrava Museum, Masarykovo náměstí 1, 728 41 Ostrava, Czech Republic

^3^ Department of Palaeontology, National Museum Prague, Václavské náměstí 68, 115 79 Prague, Czech Republic

*Corresponding author. [bartak.pavel@mail.muni.cz](mailto:bartak.pavel@mail.muni.cz), [bartak@ostrmuz.cz](mailto:bartak@ostrmuz.cz)

**The list of operational taxonomic units and the sources for the coded character states:**

*Acanthostega gunnari –* Clack (1994, 2002a, b); Ahlberg & Clack (1998); Porro *et al.* (2015).

*Crassigyrinus scoticus –* Panchen (1985); Clack (1997); Ahlberg & Clack (1998); Porro *et al.* (2023).

*Greererpeton burkemorani* – Smithson (1982); Bolt & Lombard (2001).

*Baphetes kirkbyi –* Beaumont (1977); Milner & Lindsay (1998).

*Baphetes orientalis* (adult, NMP M1388) – this study.

*Baphetes orientalis* (juvenile, NHMW-Geo-1898/0010/0042) – this study.

*Baphetes planiceps –* Beaumont (1977).

*Eucritta melanolimnetes –* Clack (1998, 2001).

*Kyrinion martilli –* Clack (2003).

*Loxomma acutirhinus –* Beaumont (1977).

*Loxomma allmanni –* Beaumont (1977).

*Loxomma lintonensis –* Romer (1930); Beaumont (1977).

*Loxomma rankini –* Beaumont (1977).

*Megalocephalus lineolatus* *–* Beaumont (1977).

*Megalocephalus pachycephalus –* Beaumont (1977); Ahlberg & Clack (1998).

*Spathicephalus mirus –* Beaumont & Smithson (1998).

*Spathicephalus marsdeni –* Smithson *et al.* (2017).

*Spathicephalus pereger –* Baird (1962).

**General skull characters**

1. **Skull longer than broad (0); as broad as long (1); or broader than long (2)** [Clack *et al.* 2016: char. 154].
2. **Preorbital snout less than 50% of total skull length (0); 50–65% of total skull length (1); at least 65% of total skull length (2)** [modified from Milner *et al.* 2009: char. 2].

*K. martilli* – char. 2(2) changed to 2(0).

*L. lintonensis* – char. 2(1) changed to 2(0).

*M. lineolatus* – char. 2(2) changed to 2(?).

*M. pachycephalus* – char. 2(2) changed to 2(1).

1. **Skull table shape: longer than broad (0); approximately square (1); shorter than broad (2)** [modified from Milner *et al.* 2009: char. 11; Clack *et al.* 2016: char. 169].

*C. scoticus* – char. 3(1) changed to 3(0).

*B. kirkbyi* – char. 3(0) changed to 3(1).

*B. orientalis* (adult) – char. 3(0) changed to 3(2).

*B. orientalis* (juv.) – char. 3(0) changed to 3(1).

*E. melanolimnetes* – char. 3(0) changed to 3(1).

*M. pachycephalus* – char. 3(0) changed to 3(1).

*S. mirus* – char. 3(0) changed to 3(2).

1. **Orbit shape round or oval (0); orbit with angle at anterolateral corner (1); emarginated margin including prefrontal, lacrimal and jugal forming antorbital fenestra (2).** [Milner *et al.* 2009: char. 3].

*C. scoticus* – char. 4(0) changed to 4(1).

1. **Antorbital vacuities absent or rudimentary (0); similar in size to orbit or not more than 20% larger (1); more than 20% larger than orbit (2)** [Milner *et al.* 2009: char. 4].

*Comment:* The antorbital fenestra is defined here as delimitated by the medially bulged jugal (or jugal-postorbital suture), laterally bulged prefrontal, and lacrimal (Beaumont & Smithson 1998), and measured along the midline. *Loxomma acutirhinus* lacks well-developed posteromedial bulging of the jugal, but the eminence is present on the anterior process of the jugal which is considered to represent the posterior limit of the antorbital fenestra in this species (Beaumont 1977).

*B. kirkbyi* – char. 5(1) changed to 5(2).

*B. orientalis* (adult) – char. 5(1) changed to 5(2).

*B. orientalis* (juv.) – char. 5(1) changed to 5(2).

*L. allmanni* – char. 5(1) changed to 5(2).

*L. lintonensis* – char. 5(1) changed to 5(2).

*S. mirus* – char. 5(1) changed to 5(2).

1. **Pineal foramen position along interparietal suture: behind midpoint (0); at the midpoint (1); anterior to midpoint (2)** [Clack *et al.* 2016: char. 166].

**Skull roof**

1. **Premaxilla anteroposterior depth ≤ 40% maximum width (0); anteroposterior depth > 40% maximum width (1)** [modified from Milner *et al.* 2009: char. 5].

*B. orientalis* (juv.) – char. 7(1) changed to 7(0).

1. **Premaxillary tooth number: up to 8 (0); 9–14 (1); 15–30 (2)** [modified from Milner *et al.* 2009: char. 22].

   *C. scoticus* – char. 8(2) changed to 8(1).
2. **Maxilla contacts quadratojugal (0); does not contact quadratojugal (1)** [Milner *et al.* 2009: char. 10].

*B. orientalis* (adult) – char. 9(?) changed to 9(1).

1. **Nasal extends anterior to the external naris (0); terminates posterior to its anterior margin (1)** [new character].
2. **Nasal–parietal length ratio less than 1.45 (0); greater than 1.45 (1)** [modified from Clack *et al.* 2016: char. 34].
3. **Median rostral (= internasal) present (0); absent (1)** [modified from Clack *et al.* 2016: char. 32].
4. **Lacrimal contributes to narial margin: absent, excluded by anterior tectal (0); present (1); absent, excluded by nasal/maxillary or prefrontal/maxillary suture (2)** [modified from Milner *et al.* 2009: char. 6; Clack *et al.* 2016: char. 24].

*A. gunnari* – char. 13(1) changed to 13(0).

*C. scoticus* – char. 13(0) changed to 13(1).

*B. kirkbyi* – char. 13(0) changed to 13(1).

*B. orientalis* (adult) – char. 13(0) changed to 13(1).

*B. orientalis* (juv.) – char. 13(0) changed to 13(1).

*E. melanolimnetes* – char. 13(0) changed to 13(1).

*K. martilli* – char. 13(1) changed to 13(2).

*L. acutirhinus* – char. 13(0) changed to 13(1).

*L. lintonensis* – char. 13(0) changed to 13(1).

*M. pachycephalus* – char. 13(1) changed to 13(2).

*S. mirus* – char. 13(0) changed to 13(1).

*S. marsdeni* – char. 13(0) changed to 13(1).

1. **Prefrontal less than three times longer than wide (0); more than (1)** [Clack *et al.* 2016: char. 53].
2. **Prefrontal/postfrontal length ratio ≤ 1.4 (0); greater than 1.4 (1)** [new character].
3. **Postfrontal–prefrontal suture present (0); absent (1)** [modified from Clack *et al.* 2016: char. 44].
4. **Jugal constricts the lateral edge of orbit: absent (0); formed by anterior process of jugal (1); formed by posteromedial process of jugal (2)** [modified from Milner *et al.* 2009: char. 7].
5. **Posteromedial bulging of jugal formed solely by jugal (0); formed by jugal and postorbital (1)** [modified from Milner *et al.* 2009: char. 8].

*A. gunnari* – char. 18(0) changed to 18(x)

*C. scoticus* – char. 18(0) changed to 18(x).

*B. orientalis* (juv.) – char. 18(1) changed to 18(0).

*E. melanolimnetes* – char. 18(0) changed to 18(x).

*K. martilli* – char. 18(0) changed to 18(x).

*L. acutirhinus* – char. 18(0) changed to 18(x).

*M. pachycephalus* – char. 18(0) changed to 18(1).

*S. mirus* – char. 18(0) changed to 18(x).

1. **Lacrimal process of jugal in anterior part: width** **≥ 50% antorbital vacuity diameter (0); < 50% (1)** [modified from Clack *et al.* 2016: char. 17].
2. **Jugal extends posterior to anterior antorbital vacuity margin (0); levelled with or anterior to anterior antorbital vacuity margin (1)** [modified from Clack *et al.* 2016: char. 21].
3. **Jugal excludes distal ramus of postorbital from orbit margin; no (0); yes (1)** [Milner *et al.* 2009: char. 9].

*B. orientalis* (adult) – char. 21(1) changed to 21(0).

1. **Postorbital-jugal contact broad (0); postorbital forms elongate and slender lateral process to connect with jugal (1)** [new character].
2. **Postorbital shape irregularly polygonal (0); broadly crescentic and narrowing to a posterior point (1)** [Clack *et al.* 2016: char. 47].
3. **Postorbital without distinct dorsomedial ramus for postfrontal (0); with incipient ramus (1); with elongate ramus (2)** [Clack *et al.* 2016: char. 46].
4. **Intertemporal present (0); intertemporal absent (1)** [Milner *et al.* 2009: char. 12].

*L. lintonensis* – char. 25(?) changed to 25(0).

1. **Intertemporal large, reaching ≥ 50% supratemporal length (0); small, reaching < 50% supratemporal length (1)** [modified from Clack *et al.* 2016: char. 14].
2. **Intertemporal markedly constricts postorbital: absent (0); present (1)** [new character].
3. **Supratemporal arrowhead-shaped (0); skull table portion of supratemporal square (1); supratemporal elongate (2)** [Milner *et al.* 2009: char. 14].

*C. scoticus* – char. 28(1) changed to 28(2).

*M. lineolatus* – char. 28(1) changed to 28(2).

1. **Pineal foramen on a raised boss (0); pineal foramen flush with skull table surface (1)** [modified from Milner et al. 2009: char. 13].

*C. scoticus* – char. 29(1) changed to 29(0).

*B. kirkbyi* – char. 29(0) changed to 29(1).

*E. melanolimnetes* – char. 29(0) changed to 29(1).

*K. martilli* – char. 29(0) changed to 29(1).

*L. allmanni* – char. 29(?) changed to 29(0).

*L. lintonensis* – char. 29(0) changed to 29(?).

*S. mirus* – char. 29(1) changed to 29(?).

1. **Postparietal longer than wide (0); wider than long (1); equant (2)** [Milner *et al.* 2009: char. 15].

*B. kikrbyi* – char. 30(1) changed to 30(2).

1. **Tabular with no posterior boss (0); tabular with posterior boss (1); tabular with blade (2)** [Milner *et al.* 2009: char. 16].

*A. gunnari* – char. 31(1) changed to 31(2).

*C. scoticus* – char. 31(1) changed to 31(2).

*L. allmanni* – char. 31(0) changed to 31(1).

1. **Tabular longer than wide (0); equant (1); wider than long (2)** [Milner *et al.* 2009: char. 17].

*C. scoticus* – char. 32(1) changed to 32(0).

*L. acutirhinus* – char. 32(2) changed to 32(0).

*L. allmanni* – char. 32(1) changed to 32(0).

*M. lineolatus* – char. 32(?) changed to 32(1).

1. **Squamosal posterodorsal margin shape: convex (0); sigmoidal (1); concave (2)** [Milner *et al.* 2009: char. 18].

*K. martilli* – char. 33(2) changed to 33(1).

1. **Squamosal suture with supratemporal position: within skull table (0); at apex of temporal embayment (1); dorsal to apex (2); ventral to apex (3)** [Clack *et al.* 2016: char. 61].
2. **Jaw suspensorium behind occiput (0); jaw suspensorium level with occiput (1)** [Milner *et al.* 2009: char. 19].

*L. allmanni* – char. 35(0) changed to 35(?).

*M. lineolatus* – char. 35(1) changed to 35(?).

**Palate**

1. **Anterior palatal fenestra/depression: double (0); single (1); absent (2)** [Milner *et al.* 2009: char. 20].
2. **Vomers as broad as long or broader (0); about twice as long as broad or longer (1)** [Clack *et al.* 2016: char. 91].
3. **Vomer fang pairs: present (0); absent (1)** [Clack *et al.* 2016: char. 106].
4. **Vomerine row of small teeth: present (0); absent (1)** [Clack *et al.* 2016: char. 109].
5. **Jugal: no alary process on palate (0); alary process on palate (1)** [Milner *et al.* 2009: char. 21].
6. **Parasphenoid cultriform process shape: biconvex (0); narrowly triangular (1); parallel-sided (2); or with proximal constriction followed by swelling (3)** [Clack *et al.* 2016: char. 76].

**Mandible**

1. **Lateral parasymphysial foramen: absent (0); present (1)** [Ahlberg & Clack 1998: char. 28].
2. **Dentary fangs: restricted to anterior region of dentary (0); distributed along the tooth row of dentary (1); absent (2)** [modified from Milner *et al.* 2009: char. 24].

*B. kirkbyi* – char. 43(?) changed to 43(0).

*E.* *melanolimnetes –* char. 43(0) changed to 43(2).

*K. martilli* – char. 43(1) changed to 43(0).

*M. lineolatus* – char. 43(0) changed to 43(?).

*S. mirus* – char. 43(0) changed to 43(2).

1. **Dentary with parasymphysial fangs internal to marginal tooth row: present (0); absent (1)** [Clack *et al.* 2016: char. 129].
2. **Dentary tooth number: 70–56 (0); 55–36 (1); less than 35 (2); more than 70 (3)** [modified from Clack *et al.* 2016: char. 130].
3. **Adsymphysial plate fang-pair (distinct from other teeth): absent (0); present (1)** [Clack *et al.* 2016: char. 141].
4. **Adsymphysial plate dentition: organised dentition aligned parallel to jaw margin (0); no dentition (1)** [modified from Clack *et al.* 2016: char. 142].

**Excluded characters:**

In large skulls, skull table ornament fine like rest of dermal surface of skull (0); skull table ornament coarse like rest of dermal surface of skull (1); in large skulls, skull table with finer ornament than rest of skull (2) (taxa represented by skulls less than 100 mm long treated as unknown.) [Milner *et al.* 2009: char. 1].

Ectopterygoid: row of three or more smaller teeth present (0); absent (1) [Milner *et al.* 2009: char. 23].

**Data matrix used in the phylogenetic analysis of Baphetoidea:**

10 20 30 40

*A. gunnari* 0000010100 0000000xxx 00021xx010 2000000000 0000010

*C. scoticus* 0001001101 0110010xxx 0001000200 2001000000 2?11100

*G. burkemorani* 000000110? 0121100xxx 0000a102?0 1200001010 2101200

*B. kirkbyi* 0112200110 1010002110 0002001212 1013021010 1000?10

*B. orientalis* (adult) 1122210?10 1?11102110 0112000211 112??????? ???????

*B. orientalis* (juv.) 0112210100 1?11002011 1112000210 01211????? ?????01

*B. planiceps* ???2??01?? ?01?????00 ?????????? ?????0101? ???????

*E. melanolimnetes* 0011010??? 0110000x1x 0000000210 01230?0??? 1?2????

*K. martilli* 00122?1?11 ?12???0x10 01??????1? ??110????? 100??01

*L. acutirhinus* 01121000?1 0010001x00 0011010202 1003021010 1??????

*L. allmanni* ??1220???? ???0002110 0112010202 10???????? ???????

*L. lintonensis* 001221??00 1?11002100 00020012?0 00010?1011 ??0?2??

*L. rankini* ??1??0???? ?????0???? ??00010?11 110?0????? ???????

*M. lineolatus* ??1220??0? ???110??10 ????1xx2?2 ?1???????? ?1?????

*M. pachycephalus* 0112201001 1021102010 00001xx212 1103011011 3?10210

*S. mirus* 0222220200 1110010x11 00001xx1?1 121302010? 102x300

*S. marsdeni* 212222???? 1?01110x11 00001xx1?1 02111????? ???????

*S. pereger* ??22?2???? ?????1???? ?0001xx1?2 11?3?????? ???????

Abbreviations: ? = missing data, a = polymorphic character, x = inapplicable character.

**References**

Ahlberg, P. E. and Clack, J. A. 1998. Lower jaws, lower tetrapods–a review based on the Devonian genus *Acanthostega*. *Transactions of the Royal Society of Edinburgh: Earth Sciences*, 89: 11–46.

Baird, D. 1962. A rhachitomous amphibian, *Spathicephalus*, from the Mississippian of Nova Scotia. *Breviora*, 157: 1–9.

Beaumont, E. H. 1977. Cranial morphology of the Loxommatidae (Amphibia: Labyrinthodontia). *Philosophical Transactions of the Royal Society of London, Series B*, 280: 29–101.

Beaumont, E. H. and Smithson, T. R. 1998. The cranial morphology and relationships of the aberrant Carboniferous amphibian *Spathicephalus mirus* Watson. *Zoological Journal of the Linnean Society*, 122: 187–209.

Bolt, J. R. and Lombard, R. E. 2001. The mandible of the primitive tetrapod *Greererpeton*, and the early evolution of the tetrapod lower jaw. *Journal of Paleontology*, 75: 1016–1042.

Clack, J. A. 1994. *Acanthostega gunnari*, a Devonian tetrapod from Greenland; the snout, palate and ventral parts of the braincase, with a discussion of their significance. *Meddelelser om Grønland*, 31: 1–24.

Clack, J. A. 1997. The Scottish Carboniferous tetrapod *Crassigyrinus scoticus* (Lydekker)—cranial anatomy and relationships. *Transactions of the Royal Society of Edinburgh: Earth Sciences*, 88: 127–142.

Clack, J. A. 1998. A new Early Carboniferous tetrapod with a *mélange* of crown-group characters. *Nature*, 394: 66–69.

Clack, J. A. 2001. *Eucritta melanolimnetes* from the Early Carboniferous of Scotland, a stem tetrapod showing a mosaic of characteristics. *Transactions of the Royal Society of Edinburgh: Earth Sciences*, 92: 75–95.

Clack, J. A. 2002a. A revised reconstruction of the dermal skull roof of *Acanthostega gunnari*, an early tetrapod from the Late Devonian. *Earth and Environmental Science Transactions of the Royal Society of Edinburgh*, 93: 163–165.

Clack, J. A. 2002b. The dermal skull roof of *Acanthostega gunnari*, an early tetrapod from the Late Devonian. *Transactions of the Royal Society of Edinburgh: Earth Sciences*, 93: 17–33.

Clack, J. A. 2003. A new baphetid (stem tetrapod) from the Upper Carboniferous of Tyne and Wear, U.K., and the evolution of the tetrapod occiput. *Canadian Journal of Earth Sciences*, 40: 483–498.

Clack, J. A., Bennett, C. E., Carpenter, D. K., Davies, S. J., Fraser, N. C., Kearsey, T. I., Marshall, J. E. A., Millward, D., Otoo, B. K. A., Reeves, E. J., Ross, A. J., Ruta, M., Smithson, K. Z., Smithson, T. R. and Walsh, S. A. 2016. Phylogenetic and environmental context of a Tournaisian tetrapod fauna. *Nature Ecology & Evolution*, 1: 0002.

Milner, A. C. and Lindsay, W. 1998. Postcranial remains of *Baphetes* and their bearing on the relationships of the Baphetidae (= Loxommatidae). *Zoological Journal of the Linnean Society*, 122: 211–235.

Milner, A. C., Milner, A. R. and Walsh, S. A. 2009. A new specimen of *Baphetes* from Nýřany, Czech Republic and the intrinsic relationships of the Baphetidae. *Acta Zoologica*, 90: 318–334.

Panchen, A. L. 1985. On the amphibian *Crassigyrinus scoticus* Watson from the Carboniferous of Scotland. *Philosophical Transactions of the Royal Society of London*, 309: 505–568.

Porro, L. B., Rayfield, E. J. and Clack, J. A. 2015. Descriptive anatomy and three-dimensional reconstruction of the skull of the early tetrapod *Acanthostega gunnari* Jarvik, 1952. *PLoS ONE*, 10: e0118882.

Porro, L. B., Rayfield, E. J. and Clack, J. A. 2023. Computed tomography and three-dimensional reconstruction of the skull of the stem tetrapod *Crassigyrinus scoticus* Watson, 1929. *Journal of Vertebrate Paleontology*, 42: e2183134.

Romer, A. S. 1930. The Pennsylvanian tetrapods of Linton, Ohio. *Bulletin of the American Museum of Natural History*, 59: 77–147.

Smithson, T. R. 1982. The cranial morphology of *Greererpeton burkemorani* Romer (Amphibia: Temnospondyli). *Zoological Journal of the Linnean Society*, 76: 29–90.

Smithson, T. R., Browne, M. A. E., Davies, S. J., Marshall, J. E. A., Millward, D., Walsh, S. A. and Clack, J. A. 2017. A new Mississippian tetrapod from Fife, Scotland, and its environmental context. *Papers in Palaeontology*, 3: 547–557.
